# Global burden of tuberculosis attributable to diet low in whole grains from 1990 to 2021, with projection to 2045

**DOI:** 10.3389/fnut.2025.1679569

**Published:** 2025-10-31

**Authors:** Xiaoning Lu, Xiaowei Lu, Miaomiao Jiang, Xiang Liu

**Affiliations:** ^1^Department of Cardiothoracic Surgery, The Affiliated Suqian First People’s Hospital of Nanjing Medical University, Suqian, Jiangsu, China; ^2^Department of Pulmonary and Critical Care Medicine, The Affiliated Suqian First People’s Hospital of Nanjing Medical University, Suqian, Jiangsu, China; ^3^Department of Cardiovascular Surgery, The First Affiliated Hospital of Nanjing Medical University, Nanjing, Jiangsu, China

**Keywords:** tuberculosis, diet low in whole grains, GBD 2021, SDI, DALYs, health inequality

## Abstract

**Background:**

This study, based on data from the Global Burden of Disease Study 2021 (GBD 2021), aims to analyze the global, regional, and national burden of tuberculosis attributable to diet low in whole grains (TB-DLWG) from 1990 to 2021, and to project trends through 2045.

**Methods:**

Using GBD 2021 data, the study assessed the burden of TB-DLWG through disability-adjusted life years (DALYs) and mortality rates. Decomposition analysis, cross-national inequality analysis, and the Nordpred model were employed to evaluate historical trends and predict future patterns.

**Results:**

In 2021, TB-DLWG accounted for 177,303.55 DALYs globally, representing an 8% decrease from 1990. The number of deaths was 5,539.13, a 12% reduction. However, the burden increased in low Socio-demographic Index (SDI) regions, particularly in Southern Sub-Saharan Africa. Cross-country inequality analysis revealed that low-SDI countries bore a heavier burden, though relative inequality showed improvement. Projections indicate that by 2045, the absolute number of DALYs and deaths may increase globally, but age-standardized DALY rates (ASDR) and age-standardized mortality rates (ASMR) are expected to gradually decline and stabilize.

**Conclusion:**

Although the global burden of TB-DLWG has generally declined, low SDI regions still face significant challenges. There is an urgent need to enhance public health resource allocation, promote whole grain consumption in low SDI regions.

## Introduction

Tuberculosis (TB), caused by *Mycobacterium tuberculosis*, is a chronic infectious disease that primarily affects the lungs but can also involve other organs (1–3). Clinically, over 80% of TB cases are pulmonary tuberculosis, typically presenting with cough and sputum lasting for ≥2 weeks, hemoptysis, low-grade fever, night sweats, and weight loss (4). In imaging, cavitary and infiltrative lesions are commonly observed in the apicoposterior segment of the upper lobe or the dorsal segment of the lower lobe (5). Extrapulmonary TB can affect lymph nodes, meninges, bones, the genitourinary system, and other organs, with varied clinical manifestations (6). According to the World Health Organization’s 2023 report, there were approximately 10.6 million new TB cases and 1.3 million deaths globally in 2022, making it the leading cause of death from a single infectious agent (7). India, Indonesia, China, and the Philippines together accounted for more than half of all global cases (8).

Multiple studies have demonstrated a significant inverse relationship between whole grain intake and the risk of death from infectious diseases (9). Among 367,000 participants in the U.S. NIH-AARP cohort, those in the highest quintile of whole-grain or cereal-fiber consumption experienced approximately 20% lower infectious-disease mortality than those in the lowest quintile (9). Meta-analyses have further confirmed that each additional 90 grams per day (about three servings) of whole grain intake is associated with a 20% reduction in the risk of infectious disease mortality (10). Therefore, increasing whole grain consumption may help reduce the risk of death from infectious and inflammation-related diseases.

Previous studies have primarily focused on the global epidemiological patterns of TB, often overlooking the burden and trends of TB attributable to diet low in whole grains (TB-DLWG) at the global, regional, and national levels. This study, based on data from the Global Burden of Disease Study 2021 (GBD 2021), systematically analyzes the disease burden of TB-DLWG in terms of disability-adjusted life years (DALYs) and deaths, and projects its future trends through 2045.

## Methods

### Data sources and disease definition

This study is based on data from the GBD 2021, which was developed by the Institute for Health Metrics and Evaluation (IHME) at the University of Washington (11, 12). The database integrates information from multiple sources, including epidemiological surveys, death registries, hospital records, published studies, and questionnaires from various countries, and systematically evaluates the quality and representativeness of these data (13). GBD 2021 employs the Cause of Death Ensemble model (CODEm) and DisMod-MR 2.1 to estimate DALY rates and mortality rates, respectively, while propagating uncertainty through a Bayesian framework (14–16). DALYs are calculated as the sum of years of life lost (YLL) and years lived with disability (YLD) (17). All results are age-standardized to the GBD world standard population and reported with 95% uncertainty intervals to assess precision. GBD 2021 covers 204 countries and territories, 811 subnational locations, 371 diseases and injuries, and 88 risk factors from 1990 to 2021, providing comprehensive health outcome indicators, including incidence, prevalence, mortality, and DALYs (18).

TB is a chronic infectious disease caused by *Mycobacterium tuberculosis*, primarily affecting the lungs (pulmonary tuberculosis) but also capable of involving other tissues and organs such as the meninges, bones, and urinary system (19). According to the International Classification of Diseases, 10th Revision (ICD-10), TB is coded as A15–A19.

### Decomposition analysis

In GBD 2021, decomposition analysis was used to quantify the relative contributions of population growth, aging, and epidemiological changes to variations in disease burden (e.g., mortality, and DALYs) (20). This method is widely applied in trend studies of various diseases to identify structural drivers of disease burden changes.

### Cross-country inequality analysis

To explore the distributional differences in TB-DLWG burden across countries with different levels of socioeconomic development, this study further introduced two indicators of cross-national health inequality (21). The Slope Index of Inequality (SII) measures the absolute difference in health indicators between countries with the lowest and highest Socio-demographic Index (SDI), reflecting the degree of absolute inequality. The Concentration Index (CI) assesses the distribution trend of health burden across countries with different SDI levels. CI > 0 indicates that the burden is concentrated in high-SDI countries, whereas CI < 0 indicates a concentration in low-SDI countries.

### Forecasting analysis

This study employed the Nordpred model to project the future burden of TB-DLWG. Based on the Age–Period–Cohort (APC) regression framework, this model separates the effects of age, period, and birth cohort (22). The Nordpred model uses Poisson regression combined with exponential smoothing techniques to model medium- and long-term trends and is widely used in disease burden forecasting analyses.

## Results

### Global and regional burden of TB-DLWG

**Figure 1:**
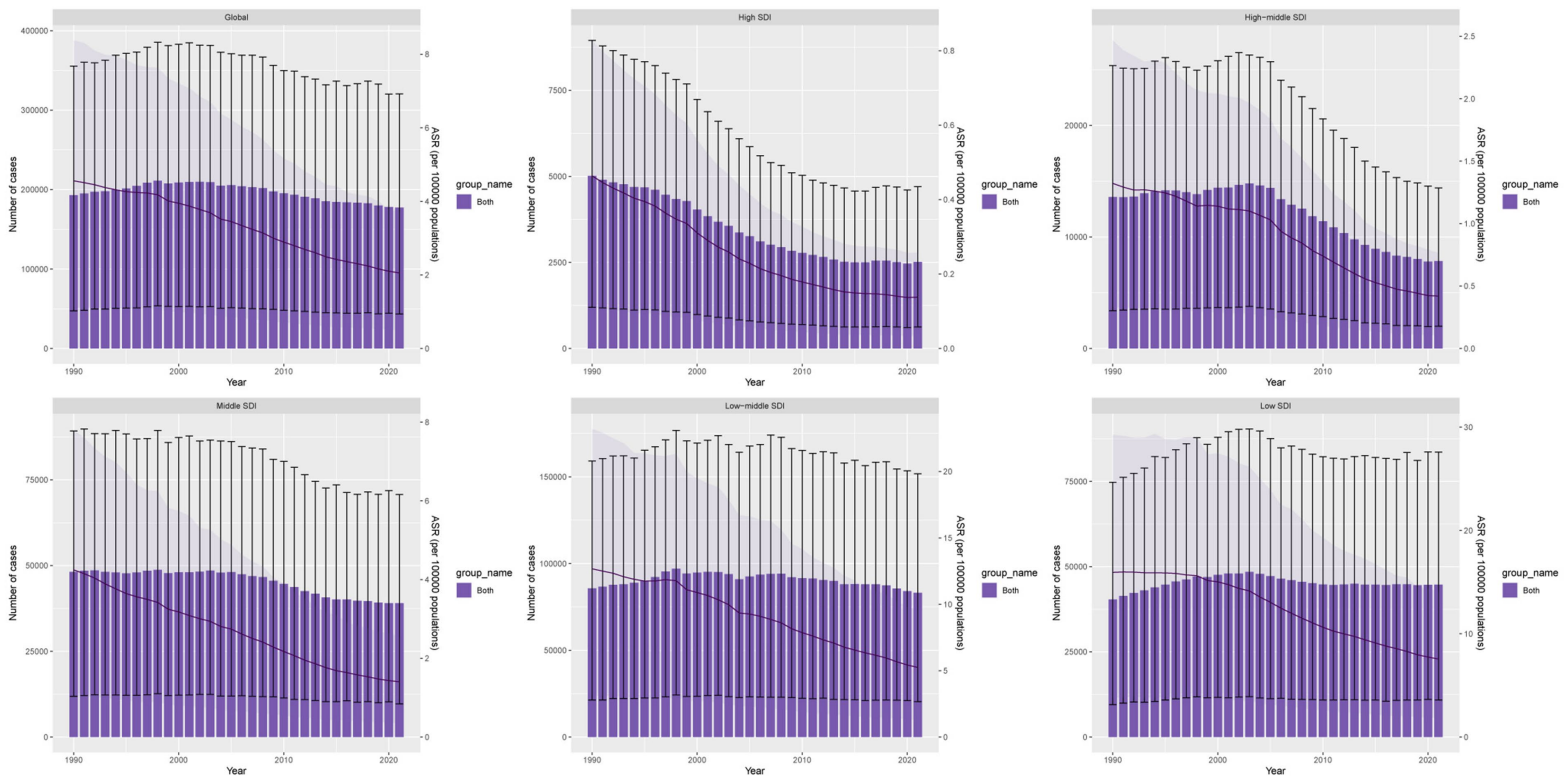
DALYs cases and ASDR of TB-DLWG from 1990 to 2021.

In 2021, the global number of DALYs due to TB-DLWG was 177,303.55, representing an 8% decrease compared to 192,931.51 in 1990. The global age-standardized DALY rate (ASDR) was 2.05 per 100,000 population, with an estimated annual percentage change (EAPC) of −2.73 (−2.88 to −2.58). The low-middle SDI regions had the highest number of DALYs cases (83,151.01). The low SDI regions were the only ones with an increase in DALYs cases, with an 11% rise. The highest ASDR was observed in the low SDI regions at 7.55 per 100,000 population. Among the 21 GBD regions, the fastest increase in DALYs cases occurred in Southern Sub-Saharan Africa, with a percentage change of 107%. The highest ASDR was recorded in South Asia (6.1 per 100,000), while the fastest growth in ASDR was also seen in Southern Sub-Saharan Africa, with an EAPC of 0.29 (−0.52 to 1.1) (Figure 1; Table 1).

**Table 1:** Disability-adjusted life years (DALYs) and age-standardized DALY rate (ASDR) of TB-DLWG in 1990 and 2021, and the PC and EAPC from 1990 to 2021.

Location	1990_DALYs cases (95% UI)	2021_DALYs cases (95% UI)	Percentage change	1990_ASDR_per 100,000 (95% UI)	2021_ASDR_per 100,000 (95% UI)	EAPC (95% CI)
Andean Latin America	907.35 (217.91–1697.38)	522.28 (125.78–985.03)	−0.42	4.07 (0.98–7.48)	0.85 (0.2–1.6)	−5.26 (−5.6 to 4.91)
Australasia	23.39 (5.79–42.37)	19.08 (4.6–33.68)	−0.18	0.1 (0.02–0.18)	0.04 (0.01–0.06)	−3.48 (−3.72 to 3.23)
Caribbean	314.81 (68.73–840.05)	325.55 (72.17–856)	0.03	1.13 (0.25–3.08)	0.62 (0.14–1.64)	−1.57 (−1.89 to 1.24)
Central Asia	579.18 (138.86–1091.66)	717.13 (167.07–1339.21)	0.24	1.1 (0.26–2.07)	0.74 (0.17–1.37)	−2.41 (−3.36 to 1.46)
Central Europe	1151.67 (282.61–2115.06)	476.42 (124.05–888.25)	−0.59	0.77 (0.19–1.42)	0.26 (0.07–0.49)	−4.03 (−4.37 to 3.68)
Central Latin America	1940.96 (480.6–3541.08)	1131.68 (269.28–2104.05)	−0.42	2.03 (0.5–3.72)	0.43 (0.1–0.8)	−5.17 (−5.63 to 4.71)
Central Sub-Saharan Africa	8816.34 (2014.42–18003.9)	14063.28 (2985.41–27364.1)	0.6	32.7 (7.49–65.27)	19.43 (4.23–37.81)	−1.87 (−2.13 to 1.61)
East Asia	25248.1 (6204.51–46780.55)	10659.8 (2598.72–19842.34)	−0.58	2.75 (0.68–5.12)	0.5 (0.12–0.93)	−5.61 (−5.81 to 5.42)
Eastern Europe	1618.52 (388.06–3076.04)	1330.66 (317.25–2454.31)	−0.18	0.58 (0.14–1.11)	0.45 (0.11–0.83)	−1.51 (−2.83 to 0.17)
Eastern Sub-Saharan Africa	10495.07 (2500.78–20030.88)	10209.97 (2401.41–19543.24)	−0.03	12.83 (3.08–24.22)	5.43 (1.29–10.18)	−3.13 (−3.28 to 2.98)
Global	192931.51 (47456.68–355523.48)	177303.55 (43303.62–320414.06)	−0.08	4.56 (1.13–8.37)	2.05 (0.5–3.72)	−2.73 (−2.88 to 2.58)
High-income Asia Pacific	2231.97 (549.2–3982.93)	1026.48 (262.07–1906.39)	−0.54	1.11 (0.27–1.97)	0.2 (0.05–0.38)	−5.84 (−6.04 to 5.64)
High-income North America	473.26 (116.48–875.4)	215.07 (54.4–379.54)	−0.55	0.14 (0.03–0.26)	0.04 (0.01–0.07)	−4.78 (−5.3 to 4.25)
High-middle SDI	13576.53 (3389.4–25374.16)	7830.86 (1999.5–14387.58)	−0.42	1.32 (0.33–2.47)	0.42 (0.11–0.76)	−4 (−4.38 to 3.62)
High SDI	5016.57 (1195.98–8949.46)	2512.47 (622.92–4702.67)	−0.5	0.46 (0.11–0.83)	0.14 (0.03–0.26)	−4.34 (−4.56 to 4.11)
Low-middle SDI	85725.81 (21388.65–159085.05)	83151.01 (20457.94–151777.46)	−0.03	12.67 (3.17–23.2)	5.24 (1.28–9.57)	−2.91 (−3.08 to 2.74)
Low SDI	40360.44 (9540.49–74741.61)	44685.46 (10858.94–83557.52)	0.11	15.94 (3.81–29.26)	7.55 (1.84–14.03)	−2.68 (−2.89 to 2.46)
Middle SDI	48163.49 (11907.9–89251.64)	39044.83 (9701.05–70729.61)	−0.19	4.25 (1.05–7.8)	1.41 (0.35–2.55)	−3.69 (−3.83 to 3.55)
North Africa and Middle East	5388.38 (1251.61–10849.02)	5590.08 (1298.94–10806.27)	0.04	2.95 (0.68–6.15)	1.03 (0.24–1.99)	−3.62 (−3.71 to 3.53)
Oceania	383.43 (88.95–720.63)	553.19 (130.71–1022.42)	0.44	10.63 (2.47–20.04)	6 (1.41–10.94)	−1.84 (−1.87 to 1.8)
South Asia	104827.24 (26987.82–192789.39)	99111.38 (24938.4–176340.08)	−0.05	16.12 (4.15–29.45)	6.1 (1.52–10.86)	−3.26 (−3.45 to 3.07)
Southeast Asia	16278.57 (3795.22–29922.51)	15480.73 (3777.47–28755.52)	−0.05	6.05 (1.41–11.03)	2.26 (0.56–4.2)	−3.32 (−3.44 to 3.2)
Southern Latin America	368.85 (88.15–694.29)	236.86 (58.91–434.43)	−0.36	0.79 (0.19–1.49)	0.29 (0.07–0.53)	−3.24 (−3.45 to 3.03)
Southern Sub-Saharan Africa	1659.96 (390.38–3197.71)	3439.05 (829.07–6402.64)	1.07	5.21 (1.23–9.98)	5.11 (1.24–9.46)	0.29 (−0.52–1.1)
Tropical Latin America	1133.44 (279.18–2102.08)	1322.02 (323.89–2370.44)	0.17	1.06 (0.26–1.97)	0.5 (0.12–0.9)	−2.61 (−2.73 to 2.49)
Western Europe	1028.16 (244.55–1872.55)	341.36 (84.99–622.33)	−0.67	0.18 (0.04–0.33)	0.04 (0.01–0.07)	−5.39 (−5.56 to 5.23)
Western Sub-Saharan Africa	8062.87 (2086.58–15043.29)	10531.48 (2615.45–19486.53)	0.31	8.71 (2.31–16.13)	4.8 (1.22–8.84)	−2.06 (−2.24 to 1.87)

In 2021, the global number of deaths due to TB-DLWG was 5,539.13, a 12% decrease from 6,287.69 in 1990. The global age-standardized mortality rate (ASMR) was 0.06453 per 100,000, with an EAPC of −3.04 (−3.2 to −2.88). The low-middle SDI regions had the highest number of deaths (2,603.81). The low SDI regions were the only ones with an increase in death cases, showing a 6% rise. The highest ASMR was observed in the low SDI regions at 0.27801 per 100,000. Among the 21 GBD regions, the fastest growth in death cases occurred in Southern Sub-Saharan Africa, with a 112% increase. The highest ASMR was found in Central Sub-Saharan Africa at 0.65268 per 100,000. The fastest-growing ASMR was in Southern Sub-Saharan Africa, with an EAPC of 0.28 (−0.48 to 1.04) (Table 2; Supplementary Figure 1).

**Table 2:** Deaths and age-standardized mortality rate (ASMR) of TB-DLWG in 1990 and 2021, and the PC and EAPC from 1990 to 2021.

Location	1990_Death cases (95% UI)	2021_Death cases (95% UI)	Percentage change	1990_ASMR_per 100,000(95% UI)	2021_ASMR_per 100,000(95% UI)	EAPC (95% CI)
Andean Latin America	31.23 (7.54–56.96)	17.98 (4.33–32.78)	−0.42	0.15357 (0.0372–0.27769)	0.03038 (0.00731–0.0553)	−5.43 (−5.76 to 5.1)
Australasia	1.06 (0.26–1.92)	0.93 (0.22–1.64)	−0.12	0.0045 (0.0011–0.00816)	0.00159 (0.00038–0.00283)	−3.61 (−3.88 to 3.35)
Caribbean	9.84 (2.16–27.69)	9.64 (2.18–26.27)	−0.02	0.03749 (0.00824–0.105)	0.01821 (0.00413–0.04978)	−1.96 (−2.27 to 1.66)
Central Asia	15.58 (3.7–28.83)	18.4 (4.3–34.08)	0.18	0.03105 (0.00739–0.05721)	0.01985 (0.00466–0.03688)	−2.47 (−3.36 to 1.57)
Central Europe	40.34 (9.98–72.85)	16.26 (4.14–29.74)	−0.6	0.02743 (0.00682–0.04938)	0.008 (0.00205–0.01466)	−4.44 (−4.76 to 4.11)
Central Latin America	61.58 (14.97–112.49)	33.5 (7.92–62.89)	−0.46	0.07294 (0.01761–0.13153)	0.01316 (0.00311–0.02475)	−5.72 (−6.16 to 5.27)
Central Sub-Saharan Africa	256.11 (57.82–510.68)	381.04 (81.21–749.11)	0.49	1.14512 (0.25914–2.26612)	0.65268 (0.14666–1.27613)	−2.03 (−2.3 to 1.75)
East Asia	848.07 (210.48–1575.91)	327.67 (78.81–632.74)	−0.61	0.10667 (0.02661–0.1941)	0.0154 (0.00369–0.02984)	−6.38 (−6.62 to 6.13)
Eastern Europe	46.73 (11.38–87.44)	37.1 (8.84–67.26)	−0.21	0.01659 (0.00402–0.03091)	0.01182 (0.00283–0.02161)	−1.69 (−2.92 to 0.44)
Eastern Sub-Saharan Africa	347.85 (83.83–646.54)	340.98 (81.45–632.79)	−0.02	0.51039 (0.12415–0.94947)	0.22539 (0.05476–0.41241)	−2.98 (−3.13 to 2.83)
Global	6287.69 (1566.68–11446.09)	5539.13 (1327.27–10136.75)	−0.12	0.15796 (0.03951–0.2862)	0.06453 (0.01545–0.11824)	−3.04 (−3.2 to 2.88)
High-income Asia Pacific	94.78 (24.17–170.12)	69.47 (18.05–128.11)	−0.27	0.04916 (0.01255–0.08811)	0.01096 (0.0028–0.02038)	−5.17 (−5.37 to 4.97)
High-income North America	19.95 (4.86–36.56)	8.19 (2.08–14.76)	−0.59	0.00561 (0.00137–0.01031)	0.0013 (0.00033–0.00232)	−5.38 (−5.93 to 4.82)
High-middle SDI	451.57 (113.83–825.02)	234.96 (57.68–439.54)	−0.48	0.04611 (0.01174–0.08382)	0.01221 (0.00299–0.02274)	−4.6 (−4.93 to 4.26)
High SDI	207.23 (50.43–365.33)	120.35 (30.47–220.21)	−0.42	0.01864 (0.00452–0.03284)	0.00536 (0.00135–0.00994)	−4.48 (−4.69 to 4.26)
Low-middle SDI	2783.43 (697.78–5065.32)	2603.81 (627.64–4709.61)	−0.06	0.46813 (0.11764–0.84247)	0.18216 (0.04375–0.32814)	−3.14 (−3.32 to 2.96)
Low SDI	1282.93 (307.02–2344.97)	1365.94 (331.32–2526.6)	0.06	0.59166 (0.14341–1.06377)	0.27801 (0.06728–0.51549)	−2.72 (−2.93 to 2.51)
Middle SDI	1559.63 (387.71–2846.5)	1211.61 (292.99–2217.92)	−0.22	0.15916 (0.03978–0.28739)	0.04578 (0.01101–0.08422)	−4.13 (−4.27 to 3.98)
North Africa and Middle East	184.01 (41.82–394.55)	166.01 (37.6–321.82)	−0.1	0.11729 (0.02611–0.26835)	0.03575 (0.0083–0.07)	−4.05 (−4.15 to 3.95)
Oceania	10.73 (2.5–20.6)	15.18 (3.51–27.75)	0.41	0.36725 (0.08509–0.69761)	0.20399 (0.04752–0.37326)	−1.88 (−1.92 to 1.84)
South Asia	3324.15 (852.91–6082.43)	3048.07 (749.59–5502.1)	−0.08	0.58787 (0.14885–1.06535)	0.20728 (0.05064–0.37506)	−3.52 (−3.72 to 3.32)
Southeast Asia	568.71 (130.63–1,033)	531.23 (130.79–977.6)	−0.07	0.24893 (0.05763–0.45457)	0.08689 (0.02158–0.16146)	−3.47 (−3.61 to 3.33)
Southern Latin America	13.19 (3.21–24.41)	8.73 (2.16–16.13)	−0.34	0.0288 (0.00703–0.05325)	0.01011 (0.0025–0.01857)	−3.36 (−3.57 to 3.16)
Southern Sub-Saharan Africa	48.46 (11.36–92.78)	102.76 (24.65–187.8)	1.12	0.17203 (0.04053–0.32865)	0.17161 (0.04109–0.31046)	0.28 (−0.48–1.04)
Tropical Latin America	32.92 (8.02–61.29)	38.96 (9.56–70.03)	0.18	0.03454 (0.00837–0.06348)	0.01498 (0.00367–0.02696)	−2.9 (−3.02 to 2.78)
Western Europe	49.4 (11.89–89.1)	19.36 (4.85–34.76)	−0.61	0.00828 (0.002–0.01492)	0.00175 (0.00044–0.00315)	−5.4 (−5.59 to 5.21)
Western Sub-Saharan Africa	283 (75.26–516.59)	347.68 (88.59–636.62)	0.23	0.3554 (0.09432–0.64755)	0.1955 (0.04967–0.3624)	−2.08 (−2.26 to 1.9)

### National burden of TB-DLWG

**Figure 2:**
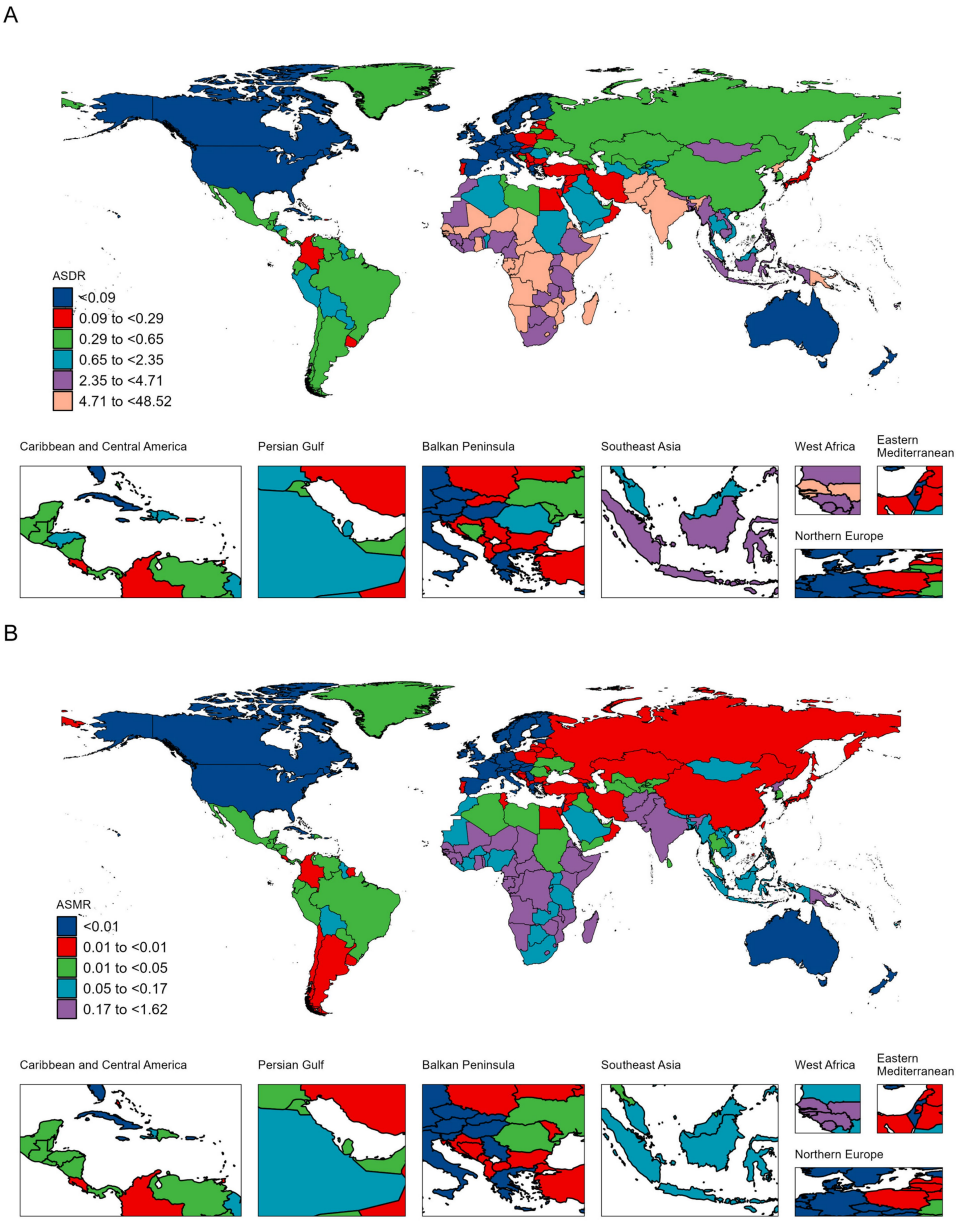
ASDR **(A)** and ASMR **(B)** of TB-DLWG per 100,000 population in 2021, by country.

Over the past 32 years, approximately 40% of countries experienced an increasing trend in DALYs cases, and around 38% of countries saw an increase in death cases. The countries with the fastest increases in DALYs cases included the United Arab Emirates, Zimbabwe, Djibouti, Kenya, and Lesotho, with percentage changes of 207, 200, 194, 187, and 184%, respectively. The fastest increases in death cases occurred in Kuwait, Djibouti, the United Arab Emirates, Zimbabwe, and Somalia, with increases of 207, 206, 173, 165, and 162%, respectively (Figure 2A; Supplementary Table 1).

Lesotho, Zimbabwe, Eswatini, Mozambique and Kenya recorded the highest ASDR increases, with EAPCs of 3.27 (2.71 to 3.84), 2.38 (1.60 to 3.17), 0.69 (−0.14 to 1.52), 0.24 (−0.03 to 0.51) and 0.21 (−0.17 to 0.60), respectively. The largest ASMR increases were seen in Lesotho, Zimbabwe, Eswatini, Somalia and South Africa, with EAPCs of 2.98 (2.42 to 3.55), 2.20 (1.50 to 2.90), 0.56 (−0.21 to 1.34), 0.19 (0.03 to 0.35) and 0.09 (−0.76 to 0.96) (Figure 2B; Supplementary Table 2).

### Age and sex differences in the burden of TB-DLWG

**Figure 3:**
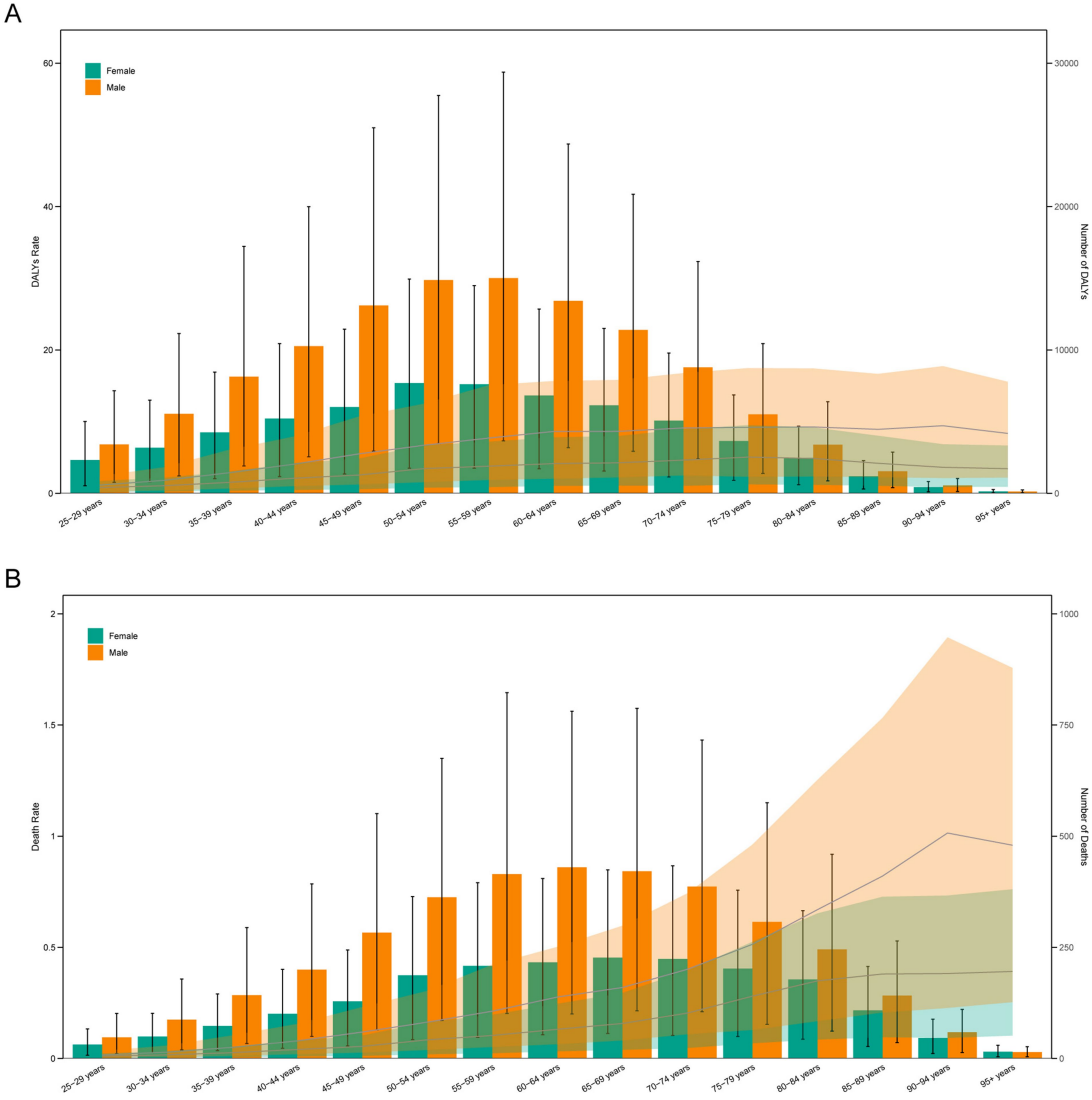
Age-specific numbers and rates of DALYs **(A)** and deaths **(B)** of TB-DLWG by age and sex in 2021.

In 2021, females aged 50–54 had the highest number of DALYs cases (7,690), while males aged 55–59 had the highest number of DALYs cases (15,015). The highest DALYs rate among females was observed in the 75–79 age group (5.06 per 100,000), while the highest DALYs rate among males was in the 90–94 age group (9.43 per 100,000) (Figure 3A). The highest number of deaths among females was in the 65–69 age group (227 cases), and among males, it was in the 60–64 age group (430 cases). The death rate among females increased with age, with the highest in the 95 + age group (0.39 per 100,000). Among males, the highest death rate was in the 90–94 age group (1.01 per 100,000) (Figure 3B).

### Relationship between the burden of TB-DLWG and SDI

**Figure 4:**
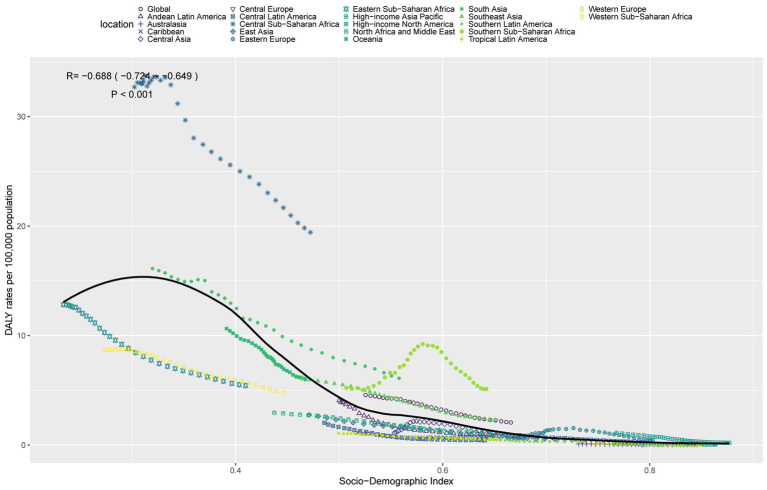
ASDR of TB-DLWG in 21 GBD regions by SDI, 1990–2021.

In 2021, ASDR and ASMR were significantly negatively correlated with SDI levels, indicating that as SDI increases, ASDR and ASMR tend to decrease (Figure 4; Supplementary Figure 2). However, some regions, including Central Sub-Saharan Africa, Southern Sub-Saharan Africa, and South Asia, experienced a disease burden significantly higher than expected, whereas regions such as Eastern Sub-Saharan Africa, Tropical Latin America, and North Africa and Middle East had a disease burden significantly lower than expected.

### Decomposition analysis of the burden of TB-DLWG

**Figure 5:**
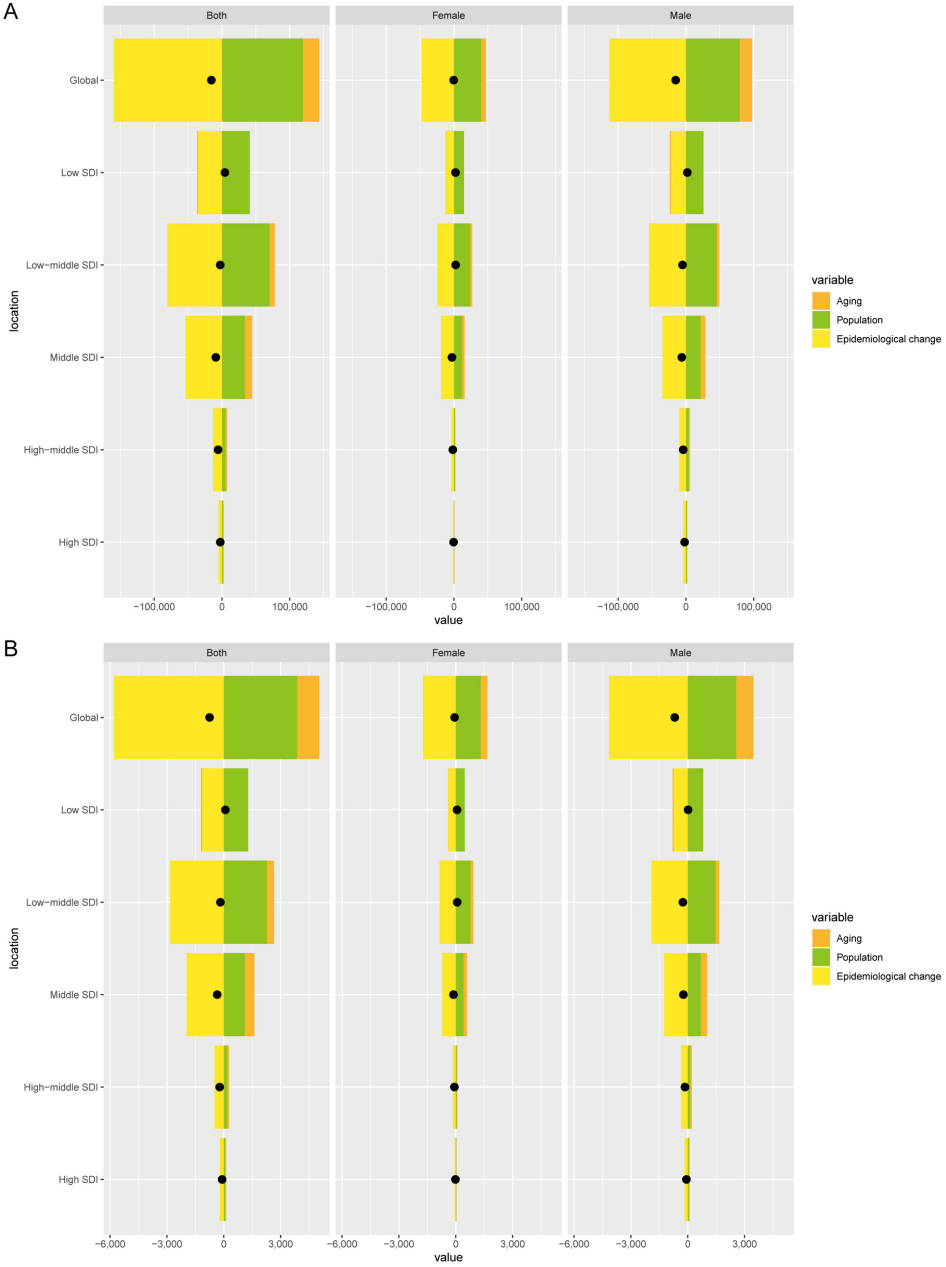
Decomposition analysis of changes in DALYs **(A)** and deaths **(B)** of TB-DLWG between 1990 and 2021 across SDI regions.

From 1990 to 2021, global DALYs cases due to TB-DLWG decreased by 15,627.96. Aging contributed 24,401.74 (−156.14%), population growth contributed 119,352.36 (−763.71%), and epidemiological change contributed −159,382.06 (1,019.85%). Among males, DALYs cases decreased by 15,168.43, with aging contributing 18,528.09 (−122.15%), population growth contributing 79,116.23 (−521.58%), and epidemiological change contributing −112,812.76 (743.73%). Among females, DALYs cases decreased by 459.53, with aging contributing 7,153.72 (−1,556.76%), population growth contributing 40,019.43 (−8,708.82%), and epidemiological change contributing −47,632.68 (10,365.58%) (Figure 5A).

Global death cases due to TB-DLWG decreased by 748.56, with aging contributing 1,175.29 (−157.01%), population growth contributing 3,868.18 (−516.75%), and epidemiological change contributing −5,792.03 (773.75%). Among males, death cases decreased by 686.36, with aging contributing 907.26 (−132.18%), population growth contributing 2,555.76 (−372.36%), and epidemiological change contributing −4,149.38 (604.55%). Among females, death cases decreased by 62.2, with aging contributing 354.26 (−569.56%), population growth contributing 1,311.86 (−2,109.13%), and epidemiological change contributing −1,728.32 (2,778.69%) (Figure 5B).

### Cross-country inequality analysis of the burden of TB-DLWG

**Figure 6:**
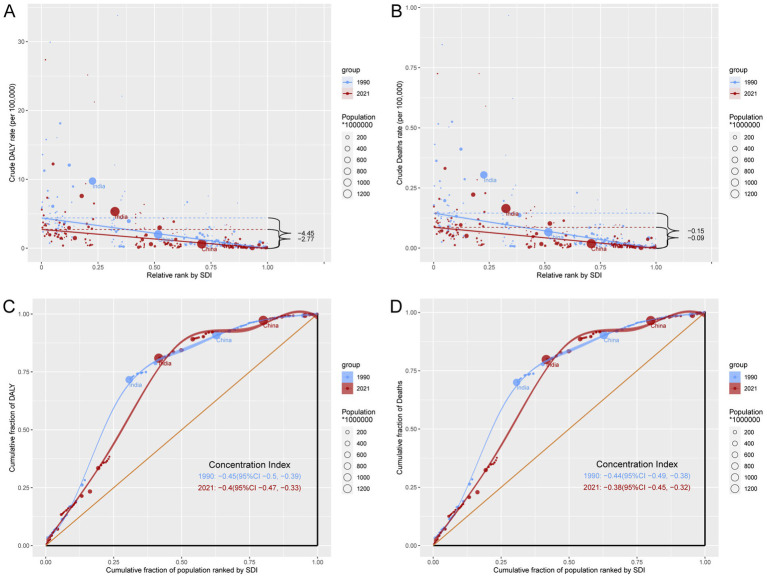
Inequality analysis of DALYs and mortality in TB-DLWG in 1990 and 2021 across the world. **(A)** Health inequality regression curves for DALYs. **(B)** Health inequality regression curves for mortality. **(C)** Concentration curves for DALYs. **(D)** Concentration curves for mortality.

Over the past 32 years, absolute and relative inequalities between countries have changed significantly. Specifically, the SII for DALYs rates changed from −4.45 in 1990 to −2.77 in 2021, and for death rates from −0.15 to −0.09, indicating that the absolute inequality between countries with the highest and lowest SDI levels has been substantially reduced (Figures 6A,B). However, the CI for DALYs rates improved slightly from −0.45 in 1990 to −0.4 in 2021, and for death rates from −0.44 to −0.38, suggesting a modest reduction in relative inequality between countries (Figures 6C,D).

### Forecast analysis of the burden of TB-DLWG

**Figure 7:**
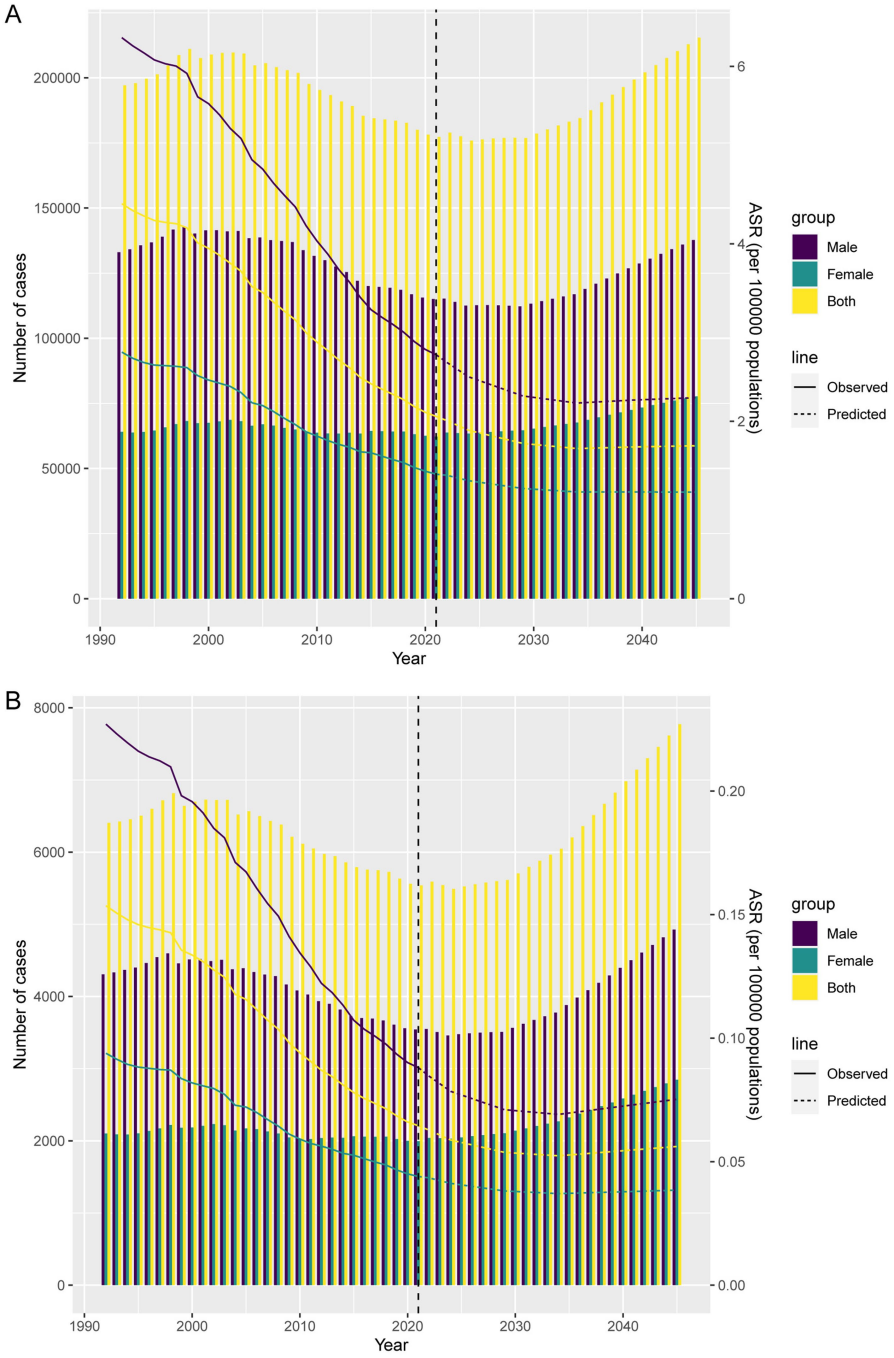
Projections of the temporal trends of the number of DALYs, mortality cases, ASDR, and ASMR of TB-DLWG globally up to 2045. **(A)** The number and ASDR of TB-DLWG by year and gender. **(B)** The number and ASMR of TB-DLWG by year and gender.

Forecast analysis indicates that from 2022 to 2045, global DALYs and death cases are expected to increase overall, while ASDR and ASMR will gradually decline and stabilize (Figures 7A,B). Specifically, by 2045, global DALYs cases will reach 215,423, with approximately 77,683 among females and 137,740 among males. The ASDR for females is projected to be about 1.2 per 100,000 and for males about 2.27 per 100,000. Global death cases are expected to reach 7,773, with 2,847 among females and 4,926 among males. The projected ASMR is approximately 0.038 per 100,000 for females and 0.075 for males.

## Discussion

From 1990 to 2021, the global burden of TB-DLWG showed an overall declining trend, with DALYs and deaths decreasing by 8 and 12%, respectively. However, DALYs and deaths increased in low SDI regions, particularly in Southern Sub-Saharan Africa. This indicates that, despite some global progress in reducing the TB-DLWG burden, low SDI regions still face severe challenges. These regional disparities may be closely linked to factors such as economic development, sanitation, nutritional intake, and public health infrastructure. Low SDI regions often lag in economic development and suffer from a shortage of healthcare resources, making it difficult to implement effective TB prevention and treatment strategies. In addition, the high prevalence of HIV in these regions further exacerbates the tuberculosis burden (23, 24).

Emerging evidence indicates that whole grain intake may influence the development and progression of TB through several biological mechanisms (25–27). Whole grains modulate immune responses by promoting regulatory T-cell differentiation, suppressing pro-inflammatory Th1/Th17 activity, and enhancing host resistance to *Mycobacterium tuberculosis*. They also reduce inflammatory cytokines (e.g., IL-22, IL-23) and regulate key pathways such as NF-κB and MAPK (25, 28). In addition, dietary fiber improves gut microbiota composition, increases beneficial metabolites like short-chain fatty acids, and strengthens intestinal barrier function, thereby supporting immune homeostasis. Whole grains further provide essential micronutrients and macronutrients that sustain immune function and facilitate recovery in TB patients (29, 30).

Nevertheless, the relationship between insufficient whole grain intake and TB burden is strongly influenced by confounding factors (31). Socioeconomic disadvantage is a major driver, as poverty, low education, and crowded living conditions increase poor dietary quality and TB susceptibility (32). Nutritional deficiencies, particularly protein–energy malnutrition, impair T-cell and macrophage activity, heighten TB risk, and worsen treatment outcomes (33). Comorbidities including HIV and diabetes further compromise nutritional status and immune function, while TB itself exacerbates malnutrition through increased metabolic demand and reduced appetite (34). Lifestyle factors (smoking, alcohol) and demographic characteristics (older age, male sex) also contribute to higher risk (35). Although whole grain intake is broadly protective against infectious diseases, TB remains primarily driven by poverty, HIV, malnutrition, and overcrowding (36). Thus, dietary factors should be interpreted within these wider social and epidemiological contexts.

Over the past 32 years, approximately 40% of countries experienced an increase in DALYs due to TB-DLWG, and about 38% of countries saw a rise in deaths. The countries with the fastest growth are mainly concentrated in Africa and the Middle East, including the United Arab Emirates, Zimbabwe, Djibouti, Kenya, and Lesotho. These regions have witnessed a significant increase in TB burden, highlighting the impact of dietary factors on TB. In terms of age and sex, our data showed that the highest number of DALYs occurred among females aged 50–54 and males aged 55–59. The highest DALYs rate in females was observed in the 75–79 age group, while for males, it peaked at 90–94 years. Regarding deaths, the highest number among females occurred in the 65–69 age group, and among males in the 60–64 age group. Mortality rates for both sexes increased significantly with age, particularly among those aged 90 and above. These data suggest that the TB burden remains high in specific populations and countries, especially among the elderly and males. In response, efforts should be made to promote whole grain intake, improve dietary patterns, and reduce TB risk associated with poor diet (37). Moreover, TB screening and treatment should be strengthened in high-burden countries and regions, particularly among older adults and men (38).

From 1990 to 2021, the global burden of TB-DLWG declined. Specifically, DALYs decreased by approximately 15,628 cases, and deaths declined by around 748 cases. These changes were primarily driven by the combined effects of population aging, growth, and epidemiological transitions. Aging and population growth contributed modestly to the increase in DALYs and deaths, but the effect of epidemiological changes was more substantial, with a large negative contribution, indicating that TB transmission and incidence have been brought under a certain degree of control.

Cross-national inequality analysis revealed significant changes in absolute and relative inequality over the past 32 years. Countries with lower socioeconomic development continue to bear a heavier disease burden, though relative inequality has decreased. This may be attributed to the redistribution of global public health resources and the widespread implementation of TB prevention and control measures.

According to projections from 2022 to 2045, the global number of DALYs and deaths due to TB-DLWG is expected to rise overall. However, ASDR and ASMR are projected to gradually decline and stabilize. By 2045, the global number of DALYs is expected to reach approximately 215,423, and deaths are projected to be around 7,773. This suggests that although the absolute number of cases may increase, advancements in public health measures and medical technology could help control the severity and fatality of the disease.

This study has several limitations. First, the analysis relied on data from the GBD 2021, in which the quality and completeness of data collection vary across countries. Estimates for low- and middle-income countries, particularly in sub-Saharan Africa and parts of Asia, may therefore be less reliable and could introduce bias. Second, the study was conducted at the population level and did not include individual-level dietary or health data. As a result, heterogeneity in individual dietary habits, lifestyle factors, and genetic predispositions could not be fully accounted for. Third, the burden of TB is shaped by multiple determinants—such as socioeconomic development, sanitation, nutrition, public health infrastructure, and HIV prevalence—yet the interactions between these factors and low whole-grain intake, as well as their independent effects, were not comprehensively assessed. Fourth, the biological mechanisms linking whole-grain intake to TB onset and progression remain only partially understood and warrant further investigation. Finally, because of the time lag in data updates within the GBD database, the findings may not fully capture the most recent epidemiological changes, which could limit the timeliness of the results.

## Conclusion

From 1990 to 2021, the global burden of TB-DLWG showed an overall declining trend. However, in low SDI regions—particularly Southern Sub-Saharan Africa—the burden of TB-DLWG significantly increased. In 2021, the highest DALYs were observed among women aged 50–54 and men aged 55–59, with mortality rates rising markedly with age. This indicates that the disease burden remains high among specific populations, such as older adults and males. Future projections suggest that from 2022 to 2045, the absolute number of DALYs and deaths due to TB-DLWG is expected to increase globally. Therefore, it is essential to enhance the global allocation of public health resources, improve sanitation and nutritional intake in low SDI regions, and promote whole grain consumption to reduce the risk of TB.

## Data Availability

The original contributions presented in the study are included in the article/Supplementary material, further inquiries can be directed to the corresponding authors.
