# Supplementary material for: Global burden of tuberculosis attributable to diet low in whole grains from 1990 to 2021, with projection to 2045
Source: Front Nutr. 2025 Oct 31;12:1679569. doi: 10.3389/fnut.2025.1679569 (PMC12616633; doi:10.3389/fnut.2025.1679569)
Supplement: Supplementary file 1 [file Table_1.pdf]

Location	1990_DALYs cases (95% UI)	2021_DALYs cases (95% UI)	Percentage change	1990_ASDR_per 100000(95% UI)	2021_ASDR_per 100 000(95% UI)	EAPC (95% CI)
Afghanistan	1568.87 (373.29-3279.57)	2287.58 (518.93-4675.67)	0.46	22.01 (5.17-46.94)	16.58 (3.81-34.14)	-1.06 (-1.27--0.85)
Albania	11.51 (3.02-23.22)	6.88 (1.6-14.66)	-0.4	0.55 (0.14-1.1)	0.17 (0.04-0.35)	-3.73 (-4.07--3.38)
Algeria	346.99 (80.98-678.31)	367.7 (81.11-731.59)	0.06	2.79 (0.63-5.46)	0.99 (0.22-1.99)	-3.41 (-3.57--3.24)
American Samoa	0.53 (0.13-0.99)	0.56 (0.14-1.02)	0.06	1.82 (0.45-3.34)	1.14 (0.29-2.1)	-1.94 (-2.16--1.71)
Andorra	0.02 (0-0.04)	0.02 (0-0.04)	0	0.03 (0.01-0.06)	0.01 (0-0.02)	-3.13 (-3.47--2.79)
Angola	764.53 (170.11-1518.41)	1132.46 (262.73-2224.53)	0.48	16.32 (3.72-31.87)	7.54 (1.8-14.32)	-2.7 (-2.88--2.51)
Antigua and Barbuda	0.11 (0.03-0.21)	0.06 (0.02-0.12)	-0.45	0.22 (0.05-0.39)	0.06 (0.02-0.11)	-4.08 (-4.44--3.72)
Argentina	218.76 (53.29-408.48)	155.26 (38.66-289.97)	-0.29	0.68 (0.17-1.26)	0.29 (0.07-0.54)	-2.69 (-2.94--2.43)
Armenia	11.02 (2.82-21.08)	6.77 (1.66-12.87)	-0.39	0.35 (0.09-0.67)	0.17 (0.04-0.33)	-2.67 (-3.7--1.62)
Australia	17.01 (4.08-30.79)	17.33 (4.16-30.68)	0.02	0.09 (0.02-0.16)	0.04 (0.01-0.07)	-2.77 (-3.06--2.48)
Austria	11.55 (2.75-21.8)	5.89 (1.47-10.91)	-0.49	0.1 (0.02-0.19)	0.03 (0.01-0.06)	-3.55 (-3.77--3.33)
Azerbaijan	74.76 (17.67-148.49)	70.73 (17.18-141.62)	-0.05	1.29 (0.3-2.56)	0.58 (0.15-1.16)	-2.85 (-3.45--2.24)
Bahamas	1.62 (0.4-2.96)	1.33 (0.33-2.47)	-0.18	0.89 (0.22-1.59)	0.3 (0.07-0.55)	-3.55 (-3.94--3.16)
Bahrain	2.84	4.86	0.71	1.38	0.52	-3.73

	(0.73-5.36)	(1.19-9.52)		(0.36-2.51)	(0.13-1.04)	(-3.89--3.57)
Bangladesh	6635.62 (1532.81-12642.56)	4870.37 (1066.02-9525.49)	-0.27	12.48 (2.89-23.69)	3.33 (0.73-6.53)	-4.08 (-4.2--3.97)
Barbados	0.76 (0.19-1.43)	0.39 (0.09-0.75)	-0.49	0.29 (0.07-0.54)	0.09 (0.02-0.17)	-4.36 (-4.76--3.95)
Belarus	35.8 (8.9-66.99)	29.72 (6.86-57.19)	-0.17	0.28 (0.07-0.53)	0.21 (0.05-0.41)	-1.49 (-2.98-0.03)
Belgium	20.45 (4.76-37.47)	8.62 (2.24-16.14)	-0.58	0.14 (0.03-0.25)	0.04 (0.01-0.07)	-4.39 (-4.56--4.22)
Belize	0.98 (0.23-1.81)	1.46 (0.33-2.78)	0.49	0.98 (0.23-1.81)	0.41 (0.09-0.77)	-3.16 (-3.56--2.76)
Benin	95.95 (23.08-186.08)	130.6 (29.46-261.87)	0.36	4.67 (1.12-8.99)	2.25 (0.52-4.47)	-2.42 (-2.57--2.28)
Bermuda	0.04 (0.01-0.08)	0.03 (0.01-0.07)	-0.25	0.07 (0.02-0.13)	0.03 (0.01-0.07)	-1.76 (-2.23--1.29)
Bhutan	24.42 (4.56-52.25)	11.7 (2.31-28.18)	-0.52	8.82 (1.71-18.77)	1.8 (0.35-4.36)	-5.33 (-5.52--5.14)
Bolivia (Plurinational State of)	272.67 (63.39-546.75)	200.89 (45.62-384.98)	-0.26	7.67 (1.81-15.4)	2.03 (0.46-3.87)	-4.52 (-4.77--4.27)
Bosnia and Herzegovina	50.01 (12.41-94.53)	16.78 (3.59-32.45)	-0.66	1.19 (0.3-2.24)	0.29 (0.06-0.56)	-4.84 (-5.13--4.55)
Botswana	58.23 (12.91-118.66)	69.73 (14.53-148.89)	0.2	9.23 (2.05-18.37)	3.89 (0.81-8.14)	-3.03 (-3.55--2.51)
Brazil	1107.46 (273.34-2055.83)	1276.41 (313.34-2306.85)	0.15	1.06 (0.26-1.98)	0.5 (0.12-0.89)	-2.65 (-2.78--2.53)
Brunei Darussalam	3.29 (0.77-6.47)	3.08 (0.76-5.73)	-0.06	2.85 (0.66-5.41)	0.9 (0.22-1.67)	-3.64 (-3.96--3.31)
Bulgaria	70.8 (18.8-132.62)	20.17 (5.23-39.34)	-0.72	0.58 (0.15-1.07)	0.17 (0.04-0.33)	-5.1 (-5.62--4.59)

Burkina Faso	225.92 (54.97-436.82)	401.01 (89.4-788.86)	0.78	5.3 (1.3-10.37)	3.95 (0.89-7.62)	-0.75 (-0.85--0.65)
Burundi	402.54 (87.12-748.46)	463.09 (109.23-914.33)	0.15	16.22 (3.54-30.24)	8.49 (2-16.6)	-2.74 (-2.97--2.5)
Cabo Verde	9.21 (2.2-18.5)	11.26 (2.48-27.05)	0.22	4.18 (0.98-8.29)	2.25 (0.5-5.37)	-2.55 (-2.78--2.31)
Cambodia	345.5 (81.57-688.2)	369.48 (84.67-739.28)	0.07	7.4 (1.78-14.76)	2.87 (0.68-5.71)	-3.56 (-3.83--3.29)
Cameroon	324.77 (76.95-644.25)	658.18 (150.5-1456.25)	1.03	7.07 (1.67-13.93)	4.52 (1.02-9.74)	-1.46 (-1.76--1.17)
Canada	18.49 (4.73-34.1)	11.01 (2.71-20.34)	-0.4	0.06 (0.01-0.1)	0.02 (0-0.03)	-4.44 (-4.85--4.02)
Central African Republic	817.07 (170.33-1633.06)	1500.98 (332.5-2959.15)	0.84	57.14 (12.11-112.88)	48.52 (10.89-92.82)	-0.63 (-0.78--0.49)
Chad	404.02 (96.55-805.3)	663.75 (146.19-1251.57)	0.64	14.15 (3.44-28.14)	10.34 (2.37-19.61)	-1.29 (-1.66--0.92)
Chile	136.01 (32.03-253.58)	72.33 (18.01-137.47)	-0.47	1.29 (0.3-2.39)	0.29 (0.07-0.56)	-4.78 (-4.99--4.56)
China	23432.62 (5756.97-43617.89)	8813.38 (2125.12-16679.15)	-0.62	2.64 (0.66-4.96)	0.43 (0.1-0.81)	-5.98 (-6.19--5.77)
Colombia	150.77 (37.79-277.58)	132.87 (30.38-244.17)	-0.12	0.77 (0.19-1.39)	0.24 (0.06-0.44)	-4.04 (-4.38--3.7)
Comoros	16.81 (3.78-32.65)	20.39 (4.83-40.65)	0.21	8.4 (1.92-16.24)	3.96 (0.94-7.89)	-2.85 (-3.12--2.57)
Congo	168.19 (32.23-330.85)	238 (54.11-479.91)	0.42	14.18 (2.75-27.77)	6.87 (1.59-13.78)	-2.72 (-2.95--2.49)
Cook Islands	0.22 (0.05-0.41)	0.14 (0.03-0.26)	-0.36	1.6 (0.36-2.95)	0.6 (0.14-1.14)	-2.96 (-3.15--2.76)
Costa Rica	14.79 (3.64-26.5)	10.61 (2.43-19.82)	-0.28	0.8 (0.2-1.44)	0.19 (0.04-0.36)	-5.31 (-5.77--

						4.85)
C 么 te d'Ivoire	351.11 (84.6-675.99)	545.03 (137.6-1108.54)	0.55	7.8 (1.88-14.73)	3.99 (1.02-8.04)	-2.35 (-2.67--2.03)
Croatia	63.53 (15.63-120.61)	10.78 (2.6-20.02)	-0.83	1.03 (0.26-1.91)	0.14 (0.03-0.26)	-6.19 (-6.4--5.99)
Cuba	13.53 (3.39-24.93)	7.97 (1.88-14.57)	-0.41	0.13 (0.03-0.24)	0.05 (0.01-0.09)	-3.37 (-3.81--2.94)
Cyprus	1.56 (0.34-3.04)	0.75 (0.19-1.39)	-0.52	0.26 (0.06-0.53)	0.04 (0.01-0.08)	-6.13 (-6.47--5.78)
Czechia	29.63 (7.94-51.97)	6.55 (1.56-12.08)	-0.78	0.22 (0.06-0.39)	0.04 (0.01-0.07)	-5.42 (-5.8--5.04)
Democratic People's Republic of Korea	1378.9 (318.37-2799.44)	1713.01 (385.39-3253.39)	0.24	8.03 (1.84-16.03)	5.08 (1.15-9.67)	-1.61 (-1.89--1.33)
Democratic Republic of the Congo	6917.67 (1549.89-14366.33)	11011.48 (2280.96-23102.7)	0.59	37.17 (8.24-76.17)	22.92 (4.87-47.44)	-1.74 (-2.02--1.46)
Denmark	4.57 (1.12-8.76)	2.48 (0.64-4.59)	-0.46	0.06 (0.01-0.11)	0.02 (0.01-0.04)	-3.5 (-3.79--3.21)
Djibouti	11.37 (2.85-23.03)	33.43 (7.84-67.13)	1.94	7.32 (1.86-14.46)	4.75 (1.14-9.24)	-1.63 (-2--1.25)
Dominica	0.71 (0.16-1.33)	0.53 (0.13-1.02)	-0.25	1.23 (0.28-2.3)	0.66 (0.16-1.26)	-1.88 (-2.26--1.49)
Dominican Republic	65.02 (16.92-131.87)	105.96 (23.42-233.62)	0.63	1.54 (0.39-3.04)	0.99 (0.22-2.17)	-0.54 (-1.04--0.05)
Ecuador	274.36 (67.46-532.82)	93.54 (24.07-171.92)	-0.66	4.8 (1.15-9.16)	0.56 (0.14-1.03)	-6.43 (-6.71--6.16)
Egypt	140.76 (33.84-265.86)	141.45 (34.33-271.56)	0	0.5 (0.12-0.93)	0.2 (0.05-0.39)	-2.44 (-2.63--2.26)
El Salvador	67.62 (16.47-126.84)	31.74 (7.37-60.28)	-0.53	2.13 (0.52-3.97)	0.52 (0.12-0.98)	-4.76 (-5.24--4.28)

Equatorial Guinea	67.86 (15.29-133.99)	65.13 (14.21-136.97)	-0.04	31.17 (7.03-60.82)	8.43 (1.91-17.51)	-4.56 (-5.23--3.88)
Eritrea	171.71 (37.67-342.52)	269.23 (57.57-575.83)	0.57	12.45 (2.89-24.15)	8.06 (1.78-16.59)	-1.56 (-1.71--1.42)
Estonia	7.52 (1.85-14.09)	2.98 (0.72-5.79)	-0.6	0.39 (0.1-0.73)	0.15 (0.04-0.29)	-4.2 (-5.37--3.01)
Eswatini	26.24 (5.64-52.3)	56.88 (12.68-114.75)	1.17	7.87 (1.74-15.57)	8.18 (1.84-16.42)	0.69 (-0.14-1.52)
Ethiopia	5695.44 (1401.9-10832.79)	2110.99 (517.62-4006.48)	-0.63	26.08 (6.48-49.22)	4.63 (1.13-8.89)	-6.23 (-6.5--5.96)
Fiji	14.84 (3.68-28.63)	19.64 (4.39-38.61)	0.32	3.42 (0.86-6.51)	2.4 (0.55-4.68)	-1.22 (-1.36--1.08)
Finland	26.24 (6.2-47.61)	5.92 (1.48-11.02)	-0.77	0.37 (0.09-0.67)	0.05 (0.01-0.09)	-6.57 (-6.72--6.41)
France	251.29 (59.4-463.09)	78.73 (19.93-145.17)	-0.69	0.3 (0.07-0.55)	0.06 (0.01-0.1)	-6.2 (-6.49--5.9)
Gabon	81.02 (17.27-163.06)	115.23 (22.75-222.54)	0.42	13.28 (2.86-26.38)	8.86 (1.76-16.98)	-1.41 (-1.77--1.05)
Gambia	27.87 (6.26-57.33)	59.54 (13.49-127.4)	1.14	7.39 (1.67-14.95)	5.44 (1.26-11.25)	-1.15 (-1.33--0.97)
Georgia	37.99 (9.44-73.09)	15.88 (4.17-29.05)	-0.58	0.61 (0.15-1.17)	0.32 (0.08-0.58)	-1.59 (-2.29--0.88)
Germany	158.64 (39.26-282.35)	41.13 (10.5-74.57)	-0.74	0.13 (0.03-0.23)	0.02 (0.01-0.04)	-5.7 (-6.17--5.22)
Ghana	623.6 (146.12-1163.74)	1443.85 (339.39-2843.06)	1.32	10.11 (2.33-18.8)	8.4 (2-16.46)	-0.2 (-0.33--0.06)
Greece	36.46 (9.87-69.93)	16.26 (4.28-30.98)	-0.55	0.24 (0.06-0.46)	0.07 (0.02-0.13)	-3.89 (-4.46--3.32)
Greenland	0.21 (0.05-0.41)	0.19 (0.04-0.35)	-0.1	0.7 (0.18-1.32)	0.29 (0.07-0.54)	-3.23 (-3.63--

						2.83)
Grenada	0.18 (0.04-0.34)	0.09 (0.02-0.16)	-0.5	0.27 (0.06-0.5)	0.07 (0.02-0.14)	-3.73 (-4.07--3.39)
Guam	2.64 (0.67-4.96)	3.33 (0.83-6)	0.26	2.96 (0.75-5.52)	1.74 (0.44-3.12)	-2.07 (-2.36--1.78)
Guatemala	136.24 (32.22-257.25)	61.77 (14.29-113.66)	-0.55	3.33 (0.78-6.31)	0.5 (0.12-0.91)	-6.66 (-7.43--5.88)
Guinea	214.22 (52.34-411.95)	272.78 (61.19-529.96)	0.27	6.38 (1.54-12.23)	4.43 (0.99-8.39)	-1.01 (-1.23--0.79)
Guinea-Bissau	61.81 (14.01-115.7)	72.84 (16.22-144.35)	0.18	14.37 (3.32-26.76)	8.43 (1.94-16.36)	-1.6 (-1.77--1.43)
Guyana	20.06 (5.35-36.36)	13.52 (2.95-26.85)	-0.33	4.58 (1.21-8.28)	1.88 (0.41-3.75)	-2.1 (-2.41--1.79)
Haiti	169.26 (31.71-649.58)	170.01 (33.69-678.14)	0	4.49 (0.84-17.9)	1.86 (0.36-7.64)	-2.64 (-2.85--2.44)
Honduras	62.27 (14.78-121.75)	79.35 (17.57-150.71)	0.27	2.67 (0.63-5.2)	1.13 (0.26-2.12)	-2.85 (-2.96--2.74)
Hungary	95.52 (23.73-173.39)	11.65 (2.93-21.8)	-0.88	0.66 (0.17-1.2)	0.07 (0.02-0.13)	-7.48 (-7.78--7.18)
Iceland	0.47 (0.11-0.86)	0.26 (0.07-0.47)	-0.45	0.16 (0.04-0.3)	0.04 (0.01-0.08)	-4.65 (-4.91--4.39)
India	83138.97 (22418-155209.13)	75213.86 (18899.42-134790.15)	-0.1	15.69 (4.22-29.37)	5.79 (1.44-10.42)	-3.33 (-3.5--3.16)
Indonesia	7258.14 (1727.41-13962.96)	8298.96 (2105.79-15066.17)	0.14	7.07 (1.66-13.27)	3.36 (0.86-6.12)	-2.42 (-2.59--2.26)
Iran (Islamic Republic of)	122.85 (27.28-238.08)	157.33 (42.15-306.64)	0.28	0.46 (0.1-0.89)	0.19 (0.05-0.36)	-2.79 (-2.98--2.6)
Iraq	484.25 (110.47-920.87)	330.44 (78.67-635.47)	-0.32	5.41 (1.26-10.14)	1.11 (0.27-2.11)	-5.62 (-5.88--5.36)
Ireland	8.19	2.92	-0.64	0.2	0.04	-5.69

	(1.93-14.97)	(0.7-5.53)		(0.05-0.37)	(0.01-0.07)	(-5.85--5.52)
Israel	8.14 (2.03-14.66)	4.01 (0.98-7.59)	-0.51	0.17 (0.04-0.31)	0.03 (0.01-0.06)	-6.29 (-6.66--5.91)
Italy	148.29 (38.01-269.46)	50.14 (13.39-92.47)	-0.66	0.17 (0.04-0.3)	0.03 (0.01-0.06)	-5.29 (-5.55--5.03)
Jamaica	1.81 (0.45-3.25)	1.19 (0.28-2.29)	-0.34	0.1 (0.03-0.19)	0.04 (0.01-0.07)	-3.19 (-3.53--2.85)
Japan	1125.99 (280.54-1995.61)	606.86 (158.26-1086.47)	-0.46	0.66 (0.16-1.17)	0.13 (0.03-0.22)	-6.01 (-6.32--5.71)
Jordan	10.71 (2.63-20.34)	17.37 (4.21-34.1)	0.62	0.72 (0.18-1.32)	0.2 (0.04-0.4)	-4.65 (-4.89--4.41)
Kazakhstan	156.32 (39.9-288.65)	85.81 (21.74-160.92)	-0.45	1.09 (0.28-2.01)	0.42 (0.11-0.8)	-5.53 (-7.21--3.82)
Kenya	615.97 (136.9-1316.82)	1769.66 (393.7-3529.5)	1.87	7.16 (1.59-15.32)	6.91 (1.56-13.59)	0.21 (-0.17-0.6)
Kiribati	25.15 (6.41-46.13)	30.49 (7.65-57.45)	0.21	55.77 (14.43-102.98)	34.78 (8.87-65.05)	-1.44 (-1.52--1.37)
Kuwait	5.45 (1.39-10.48)	12.54 (3.08-22.74)	1.3	0.62 (0.16-1.18)	0.38 (0.09-0.71)	-2.11 (-2.57--1.64)
Kyrgyzstan	30.97 (7.72-59.92)	47.03 (10.71-88.59)	0.52	0.95 (0.24-1.82)	0.78 (0.18-1.46)	-1.93 (-3.17--0.67)
Lao People's Democratic Republic	221.04 (44.48-422.62)	127.67 (28.21-248.76)	-0.42	9.94 (1.99-18.89)	2.51 (0.55-4.87)	-4.85 (-5.07--4.63)
Latvia	15.71 (3.9-30.23)	7.31 (1.71-13.81)	-0.53	0.46 (0.11-0.89)	0.25 (0.06-0.48)	-2.95 (-3.97--1.91)
Lebanon	19.07 (4.09-36.43)	13.07 (3.16-23.88)	-0.31	0.84 (0.18-1.6)	0.22 (0.05-0.4)	-4.41 (-4.63--4.2)
Lesotho	61.85 (14.75-126.32)	175.53 (41.02-365.02)	1.84	6.86 (1.62-13.98)	13.94 (3.24-28.34)	3.27 (2.71-3.84)

Liberia	89.45 (22.61-170.62)	128.87 (29.51-292.02)	0.44	7.45 (1.87-14.1)	4.52 (1.02-9.72)	-1.97 (-2.18--1.76)
Libya	15.41 (3.08-28.55)	30.93 (7.29-62.62)	1.01	0.76 (0.16-1.4)	0.49 (0.11-0.99)	-0.9 (-1.16--0.63)
Lithuania	24.94 (5.66-48.16)	13.74 (3.28-25.74)	-0.45	0.58 (0.13-1.12)	0.33 (0.08-0.62)	-2.34 (-3.39--1.29)
Luxembourg	0.5 (0.12-0.94)	0.18 (0.04-0.33)	-0.64	0.09 (0.02-0.17)	0.02 (0-0.03)	-5.81 (-5.97--5.64)
Madagascar	390.15 (92.32-792.64)	614.34 (133.18-1241.58)	0.57	7.13 (1.7-14.29)	4.82 (1.04-9.71)	-1.39 (-1.49--1.29)
Malawi	256.4 (59.21-517.41)	352.09 (76.21-738.33)	0.37	6.43 (1.54-12.7)	4.36 (0.97-8.82)	-1.72 (-2--1.43)
Malaysia	491.23 (120.7-899.19)	499.99 (123.77-957.48)	0.02	5.13 (1.25-9.34)	1.67 (0.41-3.19)	-3.93 (-4.24--3.61)
Maldives	7.16 (1.6-13.89)	2.48 (0.56-4.64)	-0.65	7.27 (1.58-14.03)	0.68 (0.16-1.27)	-8.07 (-8.47--7.66)
Mali	1177.56 (303.09-2340.74)	1148.29 (264.47-2333.13)	-0.02	26.55 (6.86-53.02)	11.19 (2.64-22.36)	-2.81 (-2.87--2.75)
Malta	0.3 (0.07-0.56)	0.13 (0.03-0.23)	-0.57	0.07 (0.02-0.13)	0.01 (0-0.03)	-5.23 (-5.43--5.02)
Marshall Islands	10.03 (2.43-19.49)	11.95 (2.76-24.33)	0.19	45.79 (10.88-88.65)	27.06 (6.38-54.85)	-1.64 (-1.77--1.5)
Mauritania	65.21 (15.08-126.45)	52.32 (12.92-103.89)	-0.2	6.45 (1.49-12.28)	2.37 (0.6-4.7)	-3.34 (-3.42--3.25)
Mauritius	7.57 (1.91-13.79)	4.79 (1.15-8.87)	-0.37	0.88 (0.22-1.59)	0.29 (0.07-0.53)	-3.64 (-3.91--3.38)
Mexico	1335.14 (338.78-2440.48)	639.11 (153.18-1232)	-0.52	2.71 (0.68-4.96)	0.47 (0.11-0.9)	-5.73 (-6.2--5.25)
Micronesia (Federated	6.5 (1.44-13.05)	3.67 (0.92-7.06)	-0.44	11.64 (2.61-23.33)	4.08 (1-7.77)	-3.68 (-3.78--

States of)						3.58)
Monaco	0.22 (0.05-0.41)	0.11 (0.02-0.2)	-0.5	0.32 (0.07-0.6)	0.12 (0.03-0.22)	-3.33 (-3.64--3.02)
Mongolia	43.25 (8.43-96.28)	92.45 (21.03-210.23)	1.14	3.49 (0.69-7.69)	2.8 (0.64-6.36)	-1.25 (-1.61--0.89)
Montenegro	2.37 (0.51-4.73)	1.73 (0.38-3.36)	-0.27	0.37 (0.08-0.73)	0.19 (0.04-0.36)	-2.48 (-2.94--2.02)
Morocco	1371.27 (288.78-3755.66)	1052.53 (240.4-2445.05)	-0.23	9.06 (1.86-26.2)	2.88 (0.67-6.75)	-3.5 (-3.82--3.17)
Mozambique	569.48 (144.86-1128.21)	1021.53 (236.28-2046.03)	0.79	9.18 (2.34-17.96)	7.94 (1.91-15.49)	0.24 (-0.03-0.51)
Myanmar	3622.83 (903.64-6817.09)	1743.21 (397.68-3358.28)	-0.52	14.26 (3.59-26.58)	3.36 (0.76-6.47)	-5.14 (-5.44--4.85)
Namibia	106.16 (25.41-211.86)	125.58 (26.89-267.32)	0.18	14.76 (3.52-29.13)	7.69 (1.66-16.52)	-2.53 (-3.07--1.98)
Nauru	0.77 (0.19-1.5)	0.55 (0.13-1.11)	-0.29	12.82 (3.08-24.89)	7.34 (1.69-14.78)	-1.97 (-2.3--1.64)
Nepal	1622.11 (358.17-3164.69)	1154.03 (294.91-2293.31)	-0.29	16.14 (3.55-31.33)	4.71 (1.19-9.49)	-4.21 (-4.31--4.11)
Netherlands	20.52 (5.21-38.37)	9 (2.22-16.57)	-0.56	0.1 (0.03-0.19)	0.03 (0.01-0.05)	-5 (-5.25--4.76)
New Zealand	6.38 (1.67-11.95)	1.75 (0.44-3.31)	-0.73	0.16 (0.04-0.31)	0.02 (0.01-0.04)	-6.4 (-7.12--5.67)
Nicaragua	34.57 (8.38-62.57)	26.49 (6.37-50.26)	-0.23	1.96 (0.48-3.53)	0.48 (0.11-0.91)	-4.99 (-5.18--4.81)
Niger	481.14 (110.09-977.66)	749.25 (161.37-1549.14)	0.56	14.65 (3.42-29.88)	7.72 (1.72-15.57)	-2.36 (-2.54--2.17)
Nigeria	3291.07 (886.8-6443.27)	3397.43 (845.03-6321.25)	0.03	7.16 (1.93-13.93)	3.41 (0.82-6.33)	-2.76 (-3.1--2.41)
Niue	0.07	0.04	-0.43	3.41	2.11	-1.77

	(0.02-0.14)	(0.01-0.08)		(0.8-6.71)	(0.52-3.91)	(-1.89--1.66)
North Macedonia	19.36 (4.59-37.03)	6.9 (1.49-13.48)	-0.64	1 (0.24-1.89)	0.22 (0.05-0.43)	-4.88 (-5.22--4.53)
Northern Mariana Islands	2.96 (0.69-5.84)	1.5 (0.39-2.83)	-0.49	10.23 (2.51-20.2)	2.77 (0.72-5.18)	-4.3 (-4.6--4)
Norway	14.53 (3.51-27.26)	7.54 (1.98-13.29)	-0.48	0.2 (0.05-0.37)	0.07 (0.02-0.12)	-3.54 (-3.88--3.2)
Oman	5.64 (1.22-11.1)	5.36 (1.22-11.16)	-0.05	0.75 (0.16-1.44)	0.2 (0.05-0.39)	-3.66 (-4.04--3.28)
Pakistan	13406.11 (3113.82-25491.89)	17861.43 (4394.75-35982.49)	0.33	22.32 (5.15-42.48)	11.93 (3-23.93)	-2.26 (-2.7--1.82)
Palau	0.51 (0.11-1)	0.61 (0.16-1.16)	0.2	4.52 (0.95-8.58)	2.63 (0.66-5.02)	-1.57 (-1.66--1.47)
Palestine	5.69 (1.26-11.12)	5.47 (1.52-10.5)	-0.04	0.66 (0.15-1.28)	0.19 (0.05-0.37)	-3.91 (-4.1--3.72)
Panama	26.67 (6.65-48.4)	22.78 (5.04-43.14)	-0.15	1.69 (0.42-3.06)	0.52 (0.11-0.98)	-3.95 (-4.25--3.66)
Papua New Guinea	273.66 (63.91-530)	428.11 (99.93-807.23)	0.56	12.59 (2.97-24.19)	6.75 (1.57-12.5)	-2 (-2.04--1.95)
Paraguay	25.98 (6.16-49.43)	45.61 (10.94-86.94)	0.76	1.08 (0.26-2.05)	0.72 (0.17-1.36)	-1.1 (-1.2--1.01)
Peru	360.32 (86.27-691.05)	227.85 (52.47-457.92)	-0.37	2.76 (0.67-5.17)	0.66 (0.15-1.33)	-5.2 (-5.91--4.49)
Philippines	1232.27 (302.88-2331.89)	2156.52 (548.94-4078.18)	0.75	3.69 (0.91-7)	2.44 (0.62-4.59)	-1.23 (-1.38--1.08)
Poland	419.13 (108.56-770.62)	121.33 (30.54-223.61)	-0.71	0.96 (0.25-1.78)	0.2 (0.05-0.37)	-5.25 (-5.6--4.9)
Portugal	87.71 (20.31-160.71)	35.16 (9.47-66.08)	-0.6	0.65 (0.15-1.19)	0.16 (0.04-0.29)	-5.25 (-5.6--4.91)

Puerto Rico	20.95 (5.04-37.25)	4.94 (1.22-9.06)	-0.76	0.59 (0.14-1.04)	0.1 (0.03-0.19)	-5.48 (-5.74--5.22)
Qatar	4.19 (0.97-8.12)	9.52 (2.29-19.08)	1.27	3.02 (0.76-5.65)	0.96 (0.22-1.79)	-4.35 (-4.78--3.92)
Republic of Korea	1070.63 (253.76-1954.86)	404.97 (96.57-737.77)	-0.62	3.59 (0.87-6.51)	0.44 (0.11-0.81)	-6.67 (-7.04--6.31)
Republic of Moldova	25.51 (6.15-49.11)	26.6 (6.21-49.11)	0.04	0.55 (0.13-1.04)	0.5 (0.11-0.92)	-0.51 (-1.87-0.87)
Romania	278.22 (65.31-552.79)	236.13 (61.68-445.97)	-0.15	1.02 (0.24-2.03)	0.78 (0.2-1.49)	-1.86 (-2.57--1.16)
Russian Federation	1116.51 (271.73-2133.81)	869.47 (202.95-1609.96)	-0.22	0.62 (0.15-1.18)	0.43 (0.1-0.8)	-1.86 (-3.26--0.44)
Rwanda	320.8 (71.22-610.55)	180.95 (43.95-360.02)	-0.44	10.56 (2.39-19.76)	2.7 (0.66-5.31)	-5.68 (-6.22--5.13)
Saint Kitts and Nevis	0.3 (0.08-0.56)	0.16 (0.04-0.3)	-0.47	0.91 (0.23-1.7)	0.22 (0.05-0.39)	-4.88 (-5.45--4.32)
Saint Lucia	1.02 (0.24-1.84)	0.71 (0.16-1.32)	-0.3	1.13 (0.27-2.03)	0.31 (0.07-0.57)	-4.33 (-4.7--3.97)
Saint Vincent and the Grenadines	0.63 (0.14-1.14)	0.32 (0.08-0.6)	-0.49	0.86 (0.19-1.56)	0.24 (0.06-0.44)	-3.88 (-4.19--3.57)
Samoa	4.91 (1.13-10.11)	5.59 (1.44-10.62)	0.14	5.11 (1.18-10.39)	3.54 (0.92-6.7)	-0.94 (-1.27--0.61)
San Marino	0.03 (0.01-0.05)	0.02 (0-0.04)	-0.33	0.07 (0.02-0.14)	0.03 (0.01-0.05)	-3.47 (-3.74--3.2)
Sao Tome and Principe	2.02 (0.49-3.98)	2.61 (0.64-5.21)	0.29	3.11 (0.75-6.03)	2.11 (0.52-4.14)	-1.5 (-1.74--1.25)
Saudi Arabia	282.89 (58.99-550.33)	502.25 (118.21-997.67)	0.78	3.81 (0.81-7.17)	1.38 (0.32-2.64)	-3.72 (-3.94--3.49)
Senegal	393.02 (95.86-739)	423.47 (109.49-810.	0.08	11.55 (2.82-21.75)	5.03 (1.29-9.44)	-2.71 (-2.82--

		61)				2.61)
Serbia	63.99 (14.13-124.71)	20.31 (5.04-38.52)	-0.68	0.55 (0.12-1.07)	0.14 (0.03-0.27)	-5.21 (-5.63--4.78)
Seychelles	1.3 (0.31-2.46)	1.12 (0.26-2.08)	-0.14	2.33 (0.56-4.34)	0.88 (0.2-1.63)	-3.84 (-4.15--3.53)
Sierra Leone	142.66 (33.4-270.04)	197.19 (47.78-406.18)	0.38	6.84 (1.66-12.8)	4.63 (1.12-9.66)	-0.92 (-1.31--0.52)
Singapore	32.07 (8.19-59.97)	11.57 (2.82-21.1)	-0.64	1.43 (0.37-2.68)	0.14 (0.03-0.25)	-7.52 (-7.71--7.33)
Slovakia	21.8 (5.64-40.58)	8.34 (1.91-15.75)	-0.62	0.37 (0.1-0.68)	0.09 (0.02-0.18)	-4.47 (-4.63--4.31)
Slovenia	7.37 (1.81-14.09)	1.94 (0.48-3.56)	-0.74	0.3 (0.07-0.58)	0.05 (0.01-0.09)	-5.82 (-6.32--5.31)
Solomon Islands	7.72 (1.78-15.2)	10.71 (2.85-20.35)	0.39	4.72 (1.11-9.2)	2.34 (0.63-4.4)	-2.34 (-2.49--2.18)
Somalia	459.59 (104.07-956.43)	1208.13 (303.16-2417.98)	1.63	16.57 (3.68-34.26)	17.42 (4.28-34.39)	-0.03 (-0.19-0.14)
South Africa	1133.88 (264.17-2208.89)	2191.63 (533.66-4058.81)	0.93	4.52 (1.07-8.71)	4.14 (1.01-7.57)	-0.04 (-0.94-0.88)
South Sudan	204.67 (44.31-416.01)	318.03 (75.04-631.17)	0.55	7.73 (1.71-15.66)	7.44 (1.78-14.7)	-0.41 (-0.61--0.22)
Spain	138.4 (33.59-258.97)	35.96 (9.11-68.26)	-0.74	0.27 (0.06-0.5)	0.04 (0.01-0.07)	-6.49 (-6.66--6.33)
Sri Lanka	171.56 (40.28-317.83)	124.15 (28.82-235.68)	-0.28	1.45 (0.35-2.64)	0.46 (0.11-0.88)	-3.96 (-4.36--3.56)
Sudan	268.78 (54.98-523.64)	204.02 (43.68-393.51)	-0.24	2.73 (0.58-5.27)	0.88 (0.19-1.68)	-3.64 (-3.74--3.55)
Suriname	2.41 (0.54-4.53)	2.35 (0.64-4.64)	-0.02	0.82 (0.19-1.52)	0.37 (0.1-0.73)	-2.84 (-3.16--2.53)
Sweden	17.64	6.05	-0.66	0.11	0.03	-4.99

	(4.31-32.57)	(1.45-11.44)		(0.03-0.19)	(0.01-0.05)	(-5.32--4.65)
Switzerland	12.96 (3.13-23.94)	3.32 (0.83-6.04)	-0.74	0.13 (0.03-0.23)	0.02 (0-0.03)	-6.17 (-6.34--6)
Syrian Arab Republic	31.24 (7.28-60.68)	25.5 (5.89-49.8)	-0.18	0.55 (0.13-1.04)	0.18 (0.04-0.35)	-3.88 (-4.23--3.53)
Taiwan	436.59 (107.37-811.71)	133.41 (33.85-239.71)	-0.69	2.82 (0.69-5.22)	0.32 (0.08-0.59)	-7.64 (-7.96--7.32)
Tajikistan	82.68 (19.37-164.35)	117.68 (27.36-222.13)	0.42	2.63 (0.62-5.12)	1.51 (0.36-2.88)	-2.47 (-2.97--1.96)
Thailand	1234.6 (303.08-2381.19)	876.72 (201.29-1756.64)	-0.29	3.53 (0.88-6.77)	0.83 (0.19-1.67)	-5 (-5.2--4.8)
Timor-Leste	22.34 (4.69-45.26)	37.51 (8.74-73.85)	0.68	7.38 (1.56-14.55)	4.29 (1-8.54)	-2.09 (-2.28--1.9)
Togo	81.99 (19.57-162.94)	173.09 (38.98-356.18)	1.11	6.53 (1.61-12.75)	4.26 (0.97-8.58)	-1.35 (-1.59--1.1)
Tokelau	0.08 (0.02-0.16)	0.03 (0.01-0.06)	-0.62	6.23 (1.43-12.07)	2.21 (0.55-4.03)	-3.46 (-3.61--3.31)
Tonga	1.7 (0.45-3.54)	1.46 (0.4-2.85)	-0.14	2.79 (0.74-5.8)	1.75 (0.48-3.4)	-1.64 (-1.8--1.48)
Trinidad and Tobago	4.49 (1.08-8.37)	3.31 (0.77-6.17)	-0.26	0.5 (0.12-0.91)	0.18 (0.04-0.33)	-3.62 (-3.94--3.3)
Tunisia	39.81 (9.15-78.47)	38.71 (8.11-80.57)	-0.03	0.78 (0.19-1.52)	0.29 (0.06-0.59)	-3.23 (-3.37--3.09)
T 眉 rkiye	552.02 (120.79-1139.57)	233.63 (60.16-447.14)	-0.58	1.54 (0.33-3.13)	0.25 (0.06-0.47)	-6.55 (-7--6.11)
Turkmenistan	27.21 (6.65-51.32)	68.19 (16.24-133.22)	1.51	1.18 (0.29-2.17)	1.35 (0.32-2.62)	-0.57 (-1.37-0.24)
Tuvalu	1.15 (0.28-2.18)	0.56 (0.14-1.02)	-0.51	15.65 (3.92-29.86)	5.04 (1.25-9.15)	-3.55 (-3.67--3.43)

Uganda	459.01 (109.32-979.61)	715.33 (166.23-1457.23)	0.56	6.78 (1.63-14.11)	4.17 (0.98-8.41)	-2.24 (-2.71--1.77)
Ukraine	392.54 (91.95-752.62)	380.84 (85.59-737.53)	-0.03	0.56 (0.13-1.08)	0.59 (0.13-1.13)	-0.4 (-1.52-0.73)
United Arab Emirates	6.13 (1.13-14.3)	18.81 (4.17-37.64)	2.07	0.83 (0.16-1.78)	0.42 (0.09-0.83)	-1.9 (-2.02--1.79)
United Kingdom	58.62 (14.91-105.03)	26.48 (6.59-47.25)	-0.55	0.07 (0.02-0.12)	0.02 (0.01-0.04)	-3.37 (-3.55--3.2)
United Republic of Tanzania	545.49 (136.98-1094.27)	867.62 (202.93-1773.56)	0.59	4.88 (1.23-9.61)	3.11 (0.72-6.3)	-1.67 (-1.91--1.44)
United States of America	454.54 (111.68-842.28)	203.86 (51.72-360.48)	-0.55	0.15 (0.04-0.28)	0.04 (0.01-0.07)	-4.78 (-5.31--4.24)
United States Virgin Islands	0.27 (0.07-0.52)	0.21 (0.05-0.41)	-0.22	0.28 (0.07-0.54)	0.15 (0.04-0.3)	-1.83 (-2.07--1.6)
Uruguay	14.07 (3.43-26.47)	9.26 (2.25-17.64)	-0.34	0.38 (0.09-0.72)	0.2 (0.05-0.38)	-1.97 (-2.33--1.61)
Uzbekistan	114.98 (26.82-219.39)	212.59 (52.2-409.19)	0.85	0.89 (0.21-1.7)	0.64 (0.16-1.23)	-1.45 (-2.43--0.45)
Vanuatu	5.51 (1.15-12.49)	9.39 (2.24-18.97)	0.7	7.45 (1.56-16.47)	4.43 (1.06-8.65)	-1.68 (-1.77--1.6)
Venezuela (Bolivarian Republic of)	112.89 (28.4-202.17)	126.96 (30.09-235.29)	0.12	1.08 (0.27-1.95)	0.41 (0.1-0.76)	-3.69 (-4.23--3.14)
Viet Nam	1639.47 (363.86-3173.79)	1216.54 (276.07-2367.58)	-0.26	4.13 (0.93-8)	1.24 (0.29-2.41)	-3.91 (-3.96--3.87)
Yemen	100.56 (18.75-206.62)	125.77 (26.64-260.09)	0.25	1.95 (0.37-3.98)	0.82 (0.18-1.66)	-3.12 (-3.37--2.87)
Zambia	368.15 (91.24-672.07)	256.3 (56.38-530.87)	-0.3	11.7 (2.92-21.44)	3.29 (0.72-6.74)	-5.38 (-6.33--4.42)
Zimbabwe	273.59 (64.49-529.	819.7 (185.94-164	2	6.19 (1.46-12.05)	9.66 (2.27-19.08)	2.38 (1.6-3.

22)	0.11)		17)

Supplementary table 1. The DALYs of TB-DLWG cases and rates in 1990 and 2021 across 204 countries, and the trends from 1990 to 2021.

Location	1990_death cases (95% UI)	2021_death cases (95% UI)	Percentage change	1990_ASMR_per 100000(95% UI)	2021_ASMR_per 100 000(95% UI)	EAPC (95% CI)
Afghanistan	51.35 (11.91-112.71)	63.9 (15.04-129.66)	0.24	0.77 (0.17-1.73)	0.58 (0.14-1.21)	-1.13 (-1.32--0.95)
Albania	0.46 (0.12-0.92)	0.32 (0.07-0.7)	-0.3	0.02 (0.01-0.05)	0.01 (0-0.02)	-3.77 (-4.15--3.38)
Algeria	12.47 (2.87-24.9)	13.59 (3.07-27.46)	0.09	0.13 (0.03-0.25)	0.04 (0.01-0.09)	-3.48 (-3.67--3.28)
American Samoa	0.01 (0-0.02)	0.02 (0-0.03)	1	0.05 (0.01-0.1)	0.03 (0.01-0.06)	-2.18 (-2.47--1.89)
Andorra	0 (0-0)	0 (0-0)	NA	0 (0-0)	0 (0-0)	-2.96 (-3.35--2.57)
Angola	22.67 (5.13-45.14)	31.65 (7.41-60.59)	0.4	0.59 (0.14-1.16)	0.26 (0.06-0.49)	-2.87 (-3.07--2.68)
Antigua and Barbuda	0 (0-0.01)	0 (0-0)	NA	0.01 (0-0.01)	0 (0-0)	-4.66 (-5.04--4.27)
Argentina	7.85 (1.88-14.66)	5.64 (1.39-10.41)	-0.28	0.02 (0.01-0.05)	0.01 (0-0.02)	-2.84 (-3.11--2.57)
Armenia	0.28 (0.07-0.52)	0.18 (0.04-0.33)	-0.36	0.01 (0-0.02)	0 (0-0.01)	-2.7 (-3.8--1.59)
Australia	0.79 (0.19-1.43)	0.86 (0.2-1.53)	0.09	0 (0-0.01)	0 (0-0)	-2.93 (-3.23--2.63)
Austria	0.56 (0.13-1.04)	0.31 (0.08-0.59)	-0.45	0 (0-0.01)	0 (0-0)	-3.43 (-3.69--3.18)
Azerbaijan	1.98 (0.46-3.91)	1.74 (0.45-3.58)	-0.12	0.04 (0.01-0.07)	0.02 (0-0.03)	-2.87 (-3.49--2.25)
Bahamas	0.05 (0.01-0.08)	0.04 (0.01-0.07)	-0.2	0.03 (0.01-0.05)	0.01 (0-0.02)	-4.01 (-4.39--3.63)
Bahrain	0.09	0.15	0.67	0.06	0.02	-3.31

	(0.02-0.16)	(0.04-0.3)		(0.02-0.11)	(0.01-0.05)	(-3.46--3.17)
Bangladesh	215.48 (50.22-410.69)	158.5 (33.37-315.3)	-0.26	0.47 (0.11-0.87)	0.12 (0.03-0.24)	-4.35 (-4.57--4.14)
Barbados	0.03 (0.01-0.05)	0.01 (0-0.03)	-0.67	0.01 (0-0.02)	0 (0-0.01)	-4.54 (-4.96--4.12)
Belarus	1.01 (0.24-1.86)	0.83 (0.19-1.6)	-0.18	0.01 (0-0.01)	0.01 (0-0.01)	-1.65 (-3.07--0.2)
Belgium	0.97 (0.22-1.78)	0.45 (0.11-0.84)	-0.54	0.01 (0-0.01)	0 (0-0)	-4.43 (-4.64--4.22)
Belize	0.03 (0.01-0.06)	0.04 (0.01-0.08)	0.33	0.04 (0.01-0.07)	0.01 (0-0.02)	-3.69 (-4.1--3.27)
Benin	3.59 (0.86-6.91)	4.24 (0.98-8.32)	0.18	0.19 (0.05-0.37)	0.09 (0.02-0.17)	-2.63 (-2.77--2.5)
Bermuda	0 (0-0)	0 (0-0)	NA	0 (0-0)	0 (0-0)	-6.19 (-6.62--5.75)
Bhutan	0.77 (0.15-1.67)	0.41 (0.08-1)	-0.47	0.34 (0.07-0.74)	0.07 (0.01-0.17)	-5.42 (-5.61--5.22)
Bolivia (Plurinational State of)	9.13 (2.16-18.14)	6.65 (1.52-12.76)	-0.27	0.29 (0.07-0.57)	0.07 (0.02-0.14)	-4.62 (-4.85--4.4)
Bosnia and Herzegovina	1.87 (0.47-3.49)	0.68 (0.15-1.33)	-0.64	0.05 (0.01-0.09)	0.01 (0-0.02)	-5.19 (-5.56--4.83)
Botswana	1.86 (0.41-3.68)	2.13 (0.44-4.45)	0.15	0.34 (0.08-0.66)	0.14 (0.03-0.28)	-3.12 (-3.58--2.66)
Brazil	32.05 (7.82-59.66)	37.55 (9.23-67.19)	0.17	0.03 (0.01-0.06)	0.01 (0-0.03)	-2.95 (-3.07--2.82)
Brunei Darussalam	0.11 (0.03-0.22)	0.11 (0.03-0.21)	0	0.12 (0.03-0.22)	0.04 (0.01-0.08)	-3.15 (-3.49--2.8)
Bulgaria	2.46 (0.62-4.61)	0.69 (0.17-1.35)	-0.72	0.02 (0.01-0.04)	0.01 (0-0.01)	-5.7 (-6.23--5.17)

Burkina Faso	8.27 (1.97-16.08)	13.35 (3.03-25.99)	0.61	0.23 (0.06-0.45)	0.16 (0.04-0.31)	-1.04 (-1.15--0.92)
Burundi	13.87 (2.98-26.07)	15.12 (3.55-29.9)	0.09	0.65 (0.14-1.22)	0.35 (0.08-0.69)	-2.63 (-2.85--2.4)
Cabo Verde	0.34 (0.08-0.66)	0.35 (0.07-0.83)	0.03	0.15 (0.04-0.29)	0.07 (0.02-0.18)	-2.87 (-3.2--2.55)
Cambodia	11.85 (2.67-23.72)	12.52 (2.92-25.23)	0.06	0.3 (0.07-0.61)	0.11 (0.03-0.23)	-3.62 (-3.9--3.34)
Cameroon	11.41 (2.71-21.96)	20.41 (4.61-44.66)	0.79	0.3 (0.07-0.56)	0.18 (0.04-0.37)	-1.73 (-2--1.46)
Canada	0.86 (0.22-1.58)	0.52 (0.13-0.97)	-0.4	0 (0-0)	0 (0-0)	-4.78 (-5.15--4.4)
Central African Republic	23.09 (4.91-45.4)	39.77 (8.79-76.71)	0.72	1.94 (0.41-3.77)	1.62 (0.37-3.03)	-0.71 (-0.85--0.57)
Chad	15.59 (3.77-30.42)	21.95 (5.05-41.48)	0.41	0.6 (0.15-1.17)	0.42 (0.1-0.79)	-1.48 (-1.83--1.12)
Chile	4.82 (1.14-8.9)	2.76 (0.69-5.2)	-0.43	0.05 (0.01-0.09)	0.01 (0-0.02)	-4.81 (-5.05--4.56)
China	783.07 (194.75-1463.35)	262.31 (62.03-516.09)	-0.67	0.1 (0.03-0.19)	0.01 (0-0.03)	-6.85 (-7.13--6.58)
Colombia	4.97 (1.25-8.98)	4.74 (1.12-8.6)	-0.05	0.03 (0.01-0.05)	0.01 (0-0.02)	-4.18 (-4.5--3.86)
Comoros	0.59 (0.13-1.14)	0.73 (0.17-1.48)	0.24	0.38 (0.09-0.71)	0.17 (0.04-0.34)	-3.01 (-3.26--2.75)
Congo	5.3 (1.02-10.43)	6.64 (1.52-13.36)	0.25	0.52 (0.1-1.02)	0.24 (0.06-0.48)	-2.89 (-3.12--2.66)
Cook Islands	0.01 (0-0.01)	0 (0-0.01)	-1	0.05 (0.01-0.09)	0.02 (0-0.03)	-3.53 (-3.69--3.36)
Costa Rica	0.55 (0.13-1)	0.35 (0.08-0.63)	-0.36	0.03 (0.01-0.06)	0.01 (0-0.01)	-5.96 (-6.43--

						5.48)
C 么 te d'Ivoire	10.89 (2.62-20.64)	16.08 (4.09-33.04)	0.48	0.31 (0.07-0.59)	0.15 (0.04-0.31)	-2.53 (-2.83--2.22)
Croatia	2.38 (0.57-4.38)	0.47 (0.11-0.87)	-0.8	0.04 (0.01-0.07)	0.01 (0-0.01)	-6.29 (-6.49--6.09)
Cuba	0.43 (0.11-0.79)	0.21 (0.05-0.38)	-0.51	0 (0-0.01)	0 (0-0)	-4.45 (-4.94--3.95)
Cyprus	0.08 (0.02-0.15)	0.04 (0.01-0.07)	-0.5	0.02 (0-0.04)	0 (0-0)	-6.4 (-6.88--5.91)
Czechia	1.13 (0.3-1.99)	0.24 (0.06-0.43)	-0.79	0.01 (0-0.01)	0 (0-0)	-5.78 (-6.17--5.38)
Democratic People's Republic of Korea	47.46 (11-95.8)	60.29 (13.84-113.22)	0.27	0.31 (0.07-0.63)	0.19 (0.04-0.35)	-1.85 (-2.12--1.57)
Democratic Republic of the Congo	200.36 (44.13-414.55)	298.27 (62.15-631.02)	0.49	1.3 (0.29-2.63)	0.77 (0.17-1.58)	-1.91 (-2.2--1.61)
Denmark	0.22 (0.05-0.41)	0.12 (0.03-0.23)	-0.45	0 (0-0)	0 (0-0)	-3.65 (-3.98--3.32)
Djibouti	0.35 (0.09-0.68)	1.07 (0.25-2.12)	2.06	0.3 (0.08-0.6)	0.2 (0.05-0.4)	-1.54 (-1.9--1.18)
Dominica	0.03 (0.01-0.05)	0.02 (0-0.03)	-0.33	0.05 (0.01-0.09)	0.02 (0.01-0.04)	-2.24 (-2.68--1.81)
Dominican Republic	2.12 (0.54-4.2)	3.3 (0.75-7.03)	0.56	0.06 (0.02-0.12)	0.03 (0.01-0.07)	-1.02 (-1.52--0.52)
Ecuador	9.65 (2.34-18.36)	3.13 (0.79-5.67)	-0.68	0.18 (0.04-0.34)	0.02 (0-0.04)	-6.74 (-7--6.47)
Egypt	4.67 (1.11-8.72)	4.2 (1.03-7.99)	-0.1	0.02 (0.01-0.04)	0.01 (0-0.01)	-2.74 (-2.97--2.5)
El Salvador	2.39 (0.59-4.37)	1.06 (0.25-2.03)	-0.56	0.08 (0.02-0.15)	0.02 (0-0.03)	-5.24 (-5.72--4.76)

Equatorial Guinea	2.13 (0.48-4.16)	1.59 (0.35-3.26)	-0.25	1.15 (0.26-2.22)	0.27 (0.06-0.54)	-5.2 (-5.87--4.53)
Eritrea	4.78 (1.08-9.4)	7.83 (1.73-16.43)	0.64	0.47 (0.11-0.93)	0.31 (0.07-0.63)	-1.42 (-1.55--1.29)
Estonia	0.2 (0.05-0.38)	0.09 (0.02-0.17)	-0.55	0.01 (0-0.02)	0 (0-0.01)	-3.93 (-5.1--2.74)
Eswatini	0.8 (0.18-1.6)	1.68 (0.37-3.36)	1.1	0.28 (0.06-0.55)	0.29 (0.07-0.56)	0.56 (-0.21-1.34)
Ethiopia	183.45 (45.17-344.14)	78.02 (19.11-147.74)	-0.57	1.03 (0.26-1.92)	0.21 (0.05-0.39)	-5.8 (-6.05--5.55)
Fiji	0.41 (0.1-0.77)	0.59 (0.13-1.17)	0.44	0.11 (0.03-0.21)	0.08 (0.02-0.16)	-1.15 (-1.34--0.95)
Finland	1.27 (0.3-2.33)	0.33 (0.08-0.61)	-0.74	0.02 (0-0.03)	0 (0-0)	-6.68 (-6.85--6.51)
France	13.76 (3.32-25.05)	5.06 (1.33-9.11)	-0.63	0.02 (0-0.03)	0 (0-0.01)	-6.4 (-6.76--6.04)
Gabon	2.57 (0.55-5.11)	3.12 (0.63-6.11)	0.21	0.45 (0.1-0.89)	0.28 (0.06-0.54)	-1.64 (-1.99--1.29)
Gambia	0.91 (0.2-1.86)	1.91 (0.44-3.91)	1.1	0.29 (0.07-0.6)	0.21 (0.05-0.44)	-1.29 (-1.46--1.12)
Georgia	1.03 (0.25-1.99)	0.47 (0.12-0.86)	-0.54	0.02 (0-0.03)	0.01 (0-0.02)	-1.51 (-2.2--0.81)
Germany	7.23 (1.78-12.78)	2.18 (0.56-3.95)	-0.7	0.01 (0-0.01)	0 (0-0)	-5.82 (-6.29--5.34)
Ghana	23.14 (5.26-43.22)	51.5 (12.14-101.46)	1.23	0.46 (0.1-0.87)	0.37 (0.09-0.73)	-0.4 (-0.54--0.25)
Greece	1.89 (0.5-3.53)	0.96 (0.25-1.87)	-0.49	0.01 (0-0.02)	0 (0-0.01)	-3.94 (-4.7--3.18)
Greenland	0.01 (0-0.02)	0.01 (0-0.02)	0	0.04 (0.01-0.07)	0.02 (0-0.03)	-3.18 (-3.6--2

						.76)
Grenada	0.01 (0-0.01)	0 (0-0)	-1	0.01 (0-0.02)	0 (0-0)	-4.24 (-4.59--3.88)
Guam	0.08 (0.02-0.14)	0.09 (0.02-0.17)	0.12	0.1 (0.03-0.19)	0.05 (0.01-0.08)	-2.99 (-3.33--2.64)
Guatemala	4.21 (0.99-7.97)	1.82 (0.42-3.34)	-0.57	0.12 (0.03-0.24)	0.02 (0-0.03)	-7.1 (-7.88--6.31)
Guinea	8.05 (1.92-15.5)	9.26 (2.09-17.5)	0.15	0.27 (0.06-0.51)	0.18 (0.04-0.34)	-1.2 (-1.43--0.98)
Guinea-Bissau	2.04 (0.47-3.77)	2.21 (0.5-4.36)	0.08	0.57 (0.14-1.08)	0.35 (0.08-0.68)	-1.55 (-1.69--1.41)
Guyana	0.63 (0.16-1.13)	0.39 (0.08-0.78)	-0.38	0.16 (0.04-0.29)	0.06 (0.01-0.11)	-2.46 (-2.74--2.18)
Haiti	5.15 (0.95-21.47)	4.89 (0.95-20.76)	-0.05	0.16 (0.03-0.71)	0.07 (0.01-0.29)	-2.74 (-2.94--2.54)
Honduras	1.99 (0.47-3.87)	2.62 (0.6-4.83)	0.32	0.1 (0.02-0.19)	0.04 (0.01-0.08)	-2.78 (-2.93--2.63)
Hungary	3.65 (0.9-6.5)	0.39 (0.1-0.74)	-0.89	0.02 (0.01-0.04)	0 (0-0)	-8.02 (-8.28--7.76)
Iceland	0.02 (0.01-0.04)	0.01 (0-0.03)	-0.5	0.01 (0-0.01)	0 (0-0)	-4.66 (-4.92--4.41)
India	2596.43 (697.22-4884.78)	2326.44 (569.81-4215.33)	-0.1	0.56 (0.15-1.03)	0.2 (0.05-0.36)	-3.53 (-3.7--3.35)
Indonesia	251.46 (58.48-475.04)	285.08 (73.81-520.91)	0.13	0.3 (0.07-0.55)	0.14 (0.04-0.26)	-2.4 (-2.59--2.21)
Iran (Islamic Republic of)	4.26 (0.94-8.26)	5.33 (1.4-10.45)	0.25	0.02 (0-0.04)	0.01 (0-0.01)	-3.13 (-3.32--2.93)
Iraq	15 (3.57-28.36)	9.14 (2.19-17.94)	-0.39	0.18 (0.04-0.34)	0.04 (0.01-0.07)	-5.72 (-6--5.45)
Ireland	0.4	0.15	-0.62	0.01 (0-0.02)	0 (0-0)	-5.72

	(0.09-0.73)	(0.04-0.28)				(-5.9--5.55)
Israel	0.4 (0.1-0.74)	0.2 (0.05-0.38)	-0.5	0.01 (0-0.02)	0 (0-0)	-6.65 (-7.06--6.24)
Italy	6.78 (1.72-12.18)	2.91 (0.75-5.39)	-0.57	0.01 (0-0.01)	0 (0-0)	-5.13 (-5.39--4.87)
Jamaica	0.07 (0.02-0.13)	0.04 (0.01-0.08)	-0.43	0 (0-0.01)	0 (0-0)	-3.45 (-3.81--3.09)
Japan	52.97 (13.4-95.14)	47.11 (12.11-84.56)	-0.11	0.03 (0.01-0.06)	0.01 (0-0.01)	-5.06 (-5.33--4.78)
Jordan	0.34 (0.08-0.64)	0.54 (0.12-1.06)	0.59	0.03 (0.01-0.05)	0.01 (0-0.02)	-4.45 (-4.66--4.24)
Kazakhstan	4.16 (1.05-7.62)	2.18 (0.55-3.96)	-0.48	0.03 (0.01-0.06)	0.01 (0-0.02)	-5.67 (-7.24--4.06)
Kenya	23.18 (5.11-50.42)	59.57 (13.69-117.57)	1.57	0.32 (0.07-0.7)	0.29 (0.07-0.56)	-0.13 (-0.42-0.17)
Kiribati	0.72 (0.19-1.34)	0.88 (0.23-1.66)	0.22	1.81 (0.48-3.36)	1.19 (0.33-2.19)	-1.18 (-1.28--1.07)
Kuwait	0.14 (0.04-0.27)	0.43 (0.1-0.77)	2.07	0.02 (0.01-0.04)	0.02 (0-0.03)	-1.3 (-1.83--0.76)
Kyrgyzstan	0.82 (0.2-1.56)	1.21 (0.28-2.24)	0.48	0.03 (0.01-0.05)	0.02 (0-0.04)	-1.98 (-3.19--0.75)
Lao People's Democratic Republic	7.34 (1.46-14.09)	4.17 (0.9-8.13)	-0.43	0.38 (0.08-0.73)	0.1 (0.02-0.19)	-4.85 (-5.06--4.64)
Latvia	0.44 (0.11-0.83)	0.22 (0.05-0.42)	-0.5	0.01 (0-0.02)	0.01 (0-0.01)	-2.9 (-3.94--1.85)
Lebanon	0.65 (0.14-1.25)	0.46 (0.11-0.86)	-0.29	0.03 (0.01-0.06)	0.01 (0-0.01)	-4.8 (-4.97--4.62)
Lesotho	2.09 (0.49-4.23)	5.33 (1.24-10.66)	1.55	0.25 (0.06-0.49)	0.47 (0.11-0.94)	2.98 (2.42-3.55)

Liberia	3.08 (0.76-5.78)	3.49 (0.81-8)	0.13	0.29 (0.07-0.55)	0.16 (0.04-0.34)	-2.43 (-2.64--2.21)
Libya	0.55 (0.12-1.02)	0.98 (0.23-2)	0.78	0.03 (0.01-0.06)	0.02 (0-0.04)	-1.02 (-1.35--0.69)
Lithuania	0.69 (0.16-1.3)	0.42 (0.1-0.8)	-0.39	0.02 (0-0.03)	0.01 (0-0.02)	-2.27 (-3.32--1.22)
Luxembourg	0.02 (0.01-0.04)	0.01 (0-0.02)	-0.5	0 (0-0.01)	0 (0-0)	-5.98 (-6.17--5.78)
Madagascar	13.11 (3.12-26.52)	18.89 (3.98-38.55)	0.44	0.29 (0.07-0.59)	0.2 (0.04-0.41)	-1.42 (-1.53--1.3)
Malawi	8.8 (2.13-17.45)	11.94 (2.65-24.37)	0.36	0.28 (0.07-0.54)	0.19 (0.04-0.37)	-1.67 (-1.91--1.43)
Malaysia	17.44 (4.23-31.48)	15.61 (3.86-29.7)	-0.1	0.2 (0.05-0.36)	0.06 (0.01-0.11)	-4.41 (-4.72--4.1)
Maldives	0.23 (0.05-0.44)	0.08 (0.02-0.15)	-0.65	0.28 (0.06-0.57)	0.03 (0.01-0.05)	-7.93 (-8.26--7.6)
Mali	37.33 (9.76-74.59)	34.78 (8.22-71.64)	-0.07	0.98 (0.26-1.91)	0.42 (0.1-0.86)	-2.86 (-2.93--2.78)
Malta	0.01 (0-0.02)	0.01 (0-0.01)	0	0 (0-0.01)	0 (0-0)	-6.05 (-6.34--5.76)
Marshall Islands	0.28 (0.07-0.55)	0.33 (0.08-0.69)	0.18	1.55 (0.37-3)	0.93 (0.22-1.89)	-1.55 (-1.68--1.43)
Mauritania	2.48 (0.57-4.79)	1.94 (0.5-3.93)	-0.22	0.28 (0.06-0.54)	0.1 (0.03-0.2)	-3.41 (-3.5--3.32)
Mauritius	0.2 (0.05-0.35)	0.11 (0.03-0.2)	-0.45	0.03 (0.01-0.05)	0.01 (0-0.01)	-4.19 (-4.51--3.88)
Mexico	41.33 (10.26-75.96)	17.2 (4.12-32.97)	-0.58	0.1 (0.02-0.17)	0.01 (0-0.02)	-6.52 (-6.99--6.05)
Micronesia (Federated	0.18 (0.04-0.36)	0.09 (0.02-0.19)	-0.5	0.34 (0.07-0.7)	0.12 (0.03-0.22)	-3.7 (-3.8--3

States of)						.61)
Monaco	0.01 (0-0.02)	0.01 (0-0.01)	0	0.01 (0-0.03)	0.01 (0-0.01)	-3.49 (-3.82--3.15)
Mongolia	1.2 (0.23-2.61)	2.28 (0.51-5.22)	0.9	0.1 (0.02-0.22)	0.07 (0.02-0.17)	-1.58 (-1.92--1.23)
Montenegro	0.08 (0.02-0.16)	0.06 (0.01-0.13)	-0.25	0.01 (0-0.02)	0.01 (0-0.01)	-2.41 (-2.85--1.98)
Morocco	49.77 (9.76-152.86)	33.68 (7.82-84.42)	-0.32	0.36 (0.07-1.14)	0.1 (0.02-0.27)	-3.76 (-4.16--3.36)
Mozambique	19.95 (5.1-38.93)	32.22 (7.62-63.28)	0.62	0.4 (0.1-0.78)	0.32 (0.08-0.62)	-0.05 (-0.29-0.2)
Myanmar	115.82 (29.64-217.55)	56.62 (12.84-106.16)	-0.51	0.53 (0.14-0.97)	0.12 (0.03-0.22)	-5.2 (-5.48--4.92)
Namibia	3.45 (0.81-6.8)	3.82 (0.83-8.2)	0.11	0.54 (0.13-1.07)	0.27 (0.06-0.57)	-2.64 (-3.18--2.1)
Nauru	0.02 (0-0.04)	0.01 (0-0.03)	-0.5	0.4 (0.1-0.78)	0.21 (0.05-0.43)	-2.29 (-2.6--1.97)
Nepal	54.2 (11.7-105.87)	38.97 (9.84-78.97)	-0.28	0.63 (0.14-1.25)	0.17 (0.04-0.35)	-4.4 (-4.5--4.3)
Netherlands	1.02 (0.26-1.85)	0.49 (0.12-0.9)	-0.52	0 (0-0.01)	0 (0-0)	-5.12 (-5.4--4.85)
New Zealand	0.27 (0.07-0.5)	0.06 (0.02-0.11)	-0.78	0.01 (0-0.01)	0 (0-0)	-7.08 (-8.01--6.15)
Nicaragua	1.13 (0.27-2.04)	0.82 (0.19-1.56)	-0.27	0.07 (0.02-0.13)	0.02 (0-0.03)	-5.26 (-5.43--5.08)
Niger	14.39 (3.37-29.55)	22.39 (5.12-45.73)	0.56	0.55 (0.13-1.13)	0.29 (0.07-0.59)	-2.38 (-2.56--2.2)
Nigeria	119.43 (32.25-229.77)	117.41 (28.03-220.04)	-0.02	0.3 (0.08-0.56)	0.15 (0.04-0.27)	-2.64 (-2.97--2.31)
Niue	0 (0-0)	0 (0-0)	NA	0.1 (0.02-0.2)	0.07	-1.52

					(0.02-0.13)	(-1.65--1.39)
North Macedonia	0.69 (0.17-1.33)	0.24 (0.05-0.48)	-0.65	0.04 (0.01-0.07)	0.01 (0-0.02)	-4.83 (-5.19--4.47)
Northern Mariana Islands	0.07 (0.02-0.15)	0.05 (0.01-0.09)	-0.29	0.35 (0.08-0.67)	0.1 (0.02-0.18)	-4.29 (-4.63--3.94)
Norway	0.81 (0.2-1.51)	0.49 (0.13-0.88)	-0.4	0.01 (0-0.02)	0 (0-0.01)	-3.13 (-3.51--2.75)
Oman	0.19 (0.04-0.36)	0.14 (0.03-0.3)	-0.26	0.03 (0.01-0.06)	0.01 (0-0.02)	-3.58 (-4.05--3.11)
Pakistan	457.27 (107.01-876.21)	523.75 (131.37-1065.13)	0.15	0.84 (0.2-1.62)	0.41 (0.1-0.82)	-2.56 (-2.98--2.15)
Palau	0.01 (0-0.03)	0.02 (0-0.03)	1	0.14 (0.03-0.27)	0.08 (0.02-0.15)	-1.54 (-1.67--1.4)
Palestine	0.22 (0.05-0.43)	0.17 (0.05-0.32)	-0.23	0.03 (0.01-0.06)	0.01 (0-0.01)	-4.14 (-4.42--3.85)
Panama	0.99 (0.24-1.76)	0.73 (0.16-1.38)	-0.26	0.07 (0.02-0.12)	0.02 (0-0.03)	-4.66 (-4.95--4.37)
Papua New Guinea	7.66 (1.79-14.94)	11.67 (2.69-21.86)	0.52	0.45 (0.11-0.88)	0.24 (0.05-0.45)	-2.03 (-2.08--1.98)
Paraguay	0.87 (0.21-1.66)	1.42 (0.34-2.68)	0.63	0.04 (0.01-0.07)	0.02 (0.01-0.04)	-1.32 (-1.43--1.21)
Peru	12.45 (3.02-23.17)	8.2 (1.93-16.65)	-0.34	0.1 (0.03-0.19)	0.02 (0.01-0.05)	-5.28 (-5.97--4.58)
Philippines	38.13 (9.35-72.7)	68.05 (17.07-127.83)	0.78	0.14 (0.03-0.26)	0.09 (0.02-0.16)	-1.3 (-1.48--1.12)
Poland	15.56 (4.08-28.14)	4.11 (1.03-7.43)	-0.74	0.04 (0.01-0.07)	0.01 (0-0.01)	-5.96 (-6.36--5.57)
Portugal	3.78 (0.91-6.87)	1.83 (0.49-3.46)	-0.52	0.03 (0.01-0.05)	0.01 (0-0.01)	-5.26 (-5.6--4.91)

Puerto Rico	0.68 (0.17-1.2)	0.17 (0.04-0.32)	-0.75	0.02 (0-0.03)	0 (0-0.01)	-6.04 (-6.3--5.77)
Qatar	0.12 (0.03-0.23)	0.28 (0.07-0.54)	1.33	0.14 (0.04-0.26)	0.05 (0.01-0.09)	-4.16 (-4.64--3.68)
Republic of Korea	40.46 (9.83-74.99)	21.75 (5.3-40.35)	-0.46	0.17 (0.04-0.31)	0.02 (0.01-0.04)	-6.26 (-6.6--5.93)
Republic of Moldova	0.65 (0.15-1.22)	0.7 (0.17-1.28)	0.08	0.01 (0-0.03)	0.01 (0-0.02)	-0.58 (-1.92-0.78)
Romania	8.13 (1.92-15.81)	7.65 (2-14.19)	-0.06	0.03 (0.01-0.06)	0.02 (0.01-0.04)	-1.84 (-2.53--1.13)
Russian Federation	31.67 (7.75-60.11)	24.16 (5.61-43.69)	-0.24	0.02 (0-0.03)	0.01 (0-0.02)	-2.02 (-3.33--0.7)
Rwanda	10.53 (2.37-19.6)	6.3 (1.51-12.34)	-0.4	0.44 (0.1-0.82)	0.12 (0.03-0.23)	-5.39 (-5.87--4.9)
Saint Kitts and Nevis	0.01 (0-0.02)	0.01 (0-0.01)	0	0.03 (0.01-0.06)	0.01 (0-0.01)	-4.83 (-5.35--4.32)
Saint Lucia	0.03 (0.01-0.06)	0.02 (0-0.04)	-0.33	0.04 (0.01-0.07)	0.01 (0-0.02)	-4.99 (-5.37--4.61)
Saint Vincent and the Grenadines	0.02 (0-0.04)	0.01 (0-0.02)	-0.5	0.03 (0.01-0.05)	0.01 (0-0.01)	-4.27 (-4.6--3.94)
Samoa	0.14 (0.03-0.27)	0.16 (0.04-0.3)	0.14	0.16 (0.04-0.31)	0.11 (0.03-0.21)	-0.91 (-1.26--0.55)
San Marino	0 (0-0)	0 (0-0)	NA	0 (0-0.01)	0 (0-0)	-3.88 (-4.21--3.56)
Sao Tome and Principe	0.08 (0.02-0.15)	0.08 (0.02-0.17)	0	0.13 (0.03-0.24)	0.08 (0.02-0.16)	-1.69 (-1.97--1.41)
Saudi Arabia	8.58 (1.84-16.04)	11.34 (2.62-22.51)	0.32	0.15 (0.03-0.28)	0.05 (0.01-0.09)	-4.18 (-4.41--3.94)
Senegal	13.63 (3.37-25.59	14.2 (3.66-26.85	0.04	0.46 (0.12-0.87)	0.2 (0.05-0.38)	-2.81 (-2.94--

	)	)				2.69)
Serbia	2.22 (0.48-4.37)	0.77 (0.19-1.46)	-0.65	0.02 (0-0.04)	0 (0-0.01)	-5.6 (-6.07--5.13)
Seychelles	0.04 (0.01-0.07)	0.03 (0.01-0.06)	-0.25	0.07 (0.02-0.13)	0.02 (0.01-0.05)	-3.96 (-4.25--3.66)
Sierra Leone	5.42 (1.35-9.96)	6.37 (1.55-13.23)	0.18	0.29 (0.07-0.54)	0.18 (0.04-0.38)	-1.18 (-1.56--0.8)
Singapore	1.23 (0.32-2.25)	0.5 (0.12-0.91)	-0.59	0.06 (0.02-0.11)	0.01 (0-0.01)	-7.44 (-7.68--7.19)
Slovakia	0.79 (0.21-1.44)	0.31 (0.07-0.58)	-0.61	0.01 (0-0.02)	0 (0-0.01)	-4.59 (-4.73--4.46)
Slovenia	0.27 (0.07-0.51)	0.08 (0.02-0.15)	-0.7	0.01 (0-0.02)	0 (0-0)	-5.73 (-6.24--5.23)
Solomon Islands	0.21 (0.05-0.42)	0.26 (0.07-0.5)	0.24	0.16 (0.04-0.29)	0.07 (0.02-0.13)	-2.68 (-2.8--2.56)
Somalia	14.22 (3.14-30.03)	37.3 (9.14-75.16)	1.62	0.68 (0.15-1.43)	0.76 (0.19-1.55)	0.19 (0.03-0.35)
South Africa	30.83 (7.32-59.6)	64.85 (15.87-117.53)	1.1	0.14 (0.03-0.27)	0.14 (0.03-0.25)	0.09 (-0.76-0.96)
South Sudan	7.27 (1.63-14.9)	10.47 (2.51-20.8)	0.44	0.32 (0.07-0.65)	0.31 (0.08-0.62)	-0.4 (-0.59--0.21)
Spain	5.88 (1.41-10.94)	1.97 (0.5-3.66)	-0.66	0.01 (0-0.02)	0 (0-0)	-6.21 (-6.39--6.03)
Sri Lanka	5.46 (1.32-10)	3.66 (0.8-7.07)	-0.33	0.05 (0.01-0.1)	0.01 (0-0.03)	-4.39 (-4.74--4.04)
Sudan	9.54 (1.98-18.82)	6.46 (1.35-12.72)	-0.32	0.11 (0.02-0.22)	0.03 (0.01-0.07)	-3.83 (-3.93--3.72)
Suriname	0.07 (0.02-0.13)	0.06 (0.02-0.12)	-0.14	0.02 (0.01-0.05)	0.01 (0-0.02)	-3.26 (-3.59--2.94)
Sweden	1.06	0.38	-0.64	0.01 (0-0.01)	0 (0-0)	-5.12

	(0.26-1.98)	(0.1-0.72)				(-5.52--4.71)
Switzerland	0.61 (0.15-1.12)	0.18 (0.05-0.32)	-0.7	0.01 (0-0.01)	0 (0-0)	-6.23 (-6.44--6.02)
Syrian Arab Republic	1.03 (0.24-1.98)	0.79 (0.18-1.5)	-0.23	0.02 (0.01-0.04)	0.01 (0-0.01)	-4.12 (-4.41--3.82)
Taiwan	17.53 (4.27-32.11)	5.06 (1.27-9.12)	-0.71	0.13 (0.03-0.24)	0.01 (0-0.02)	-8.43 (-8.79--8.07)
Tajikistan	2.31 (0.53-4.5)	3.19 (0.76-6.11)	0.38	0.08 (0.02-0.15)	0.05 (0.01-0.09)	-2.19 (-2.7--1.68)
Thailand	48.31 (12.38-92.88)	35.41 (8.2-70.25)	-0.27	0.16 (0.04-0.3)	0.03 (0.01-0.07)	-5.33 (-5.49--5.17)
Timor-Leste	0.74 (0.16-1.48)	1.4 (0.32-2.77)	0.89	0.32 (0.07-0.62)	0.18 (0.04-0.35)	-2.26 (-2.44--2.09)
Togo	2.94 (0.72-5.8)	5.75 (1.34-11.64)	0.96	0.29 (0.07-0.57)	0.18 (0.04-0.37)	-1.45 (-1.69--1.22)
Tokelau	0 (0-0)	0 (0-0)	NA	0.2 (0.04-0.39)	0.06 (0.02-0.11)	-3.88 (-4.07--3.68)
Tonga	0.05 (0.01-0.1)	0.04 (0.01-0.08)	-0.2	0.08 (0.02-0.17)	0.05 (0.01-0.1)	-1.67 (-1.81--1.53)
Trinidad and Tobago	0.14 (0.03-0.27)	0.1 (0.02-0.19)	-0.29	0.02 (0-0.03)	0.01 (0-0.01)	-4.14 (-4.5--3.78)
Tunisia	1.48 (0.36-2.86)	1.47 (0.3-3.04)	-0.01	0.03 (0.01-0.06)	0.01 (0-0.02)	-3.34 (-3.49--3.19)
T 眉 rkiye	19.86 (4.19-39.98)	8.2 (2.2-15.89)	-0.59	0.06 (0.01-0.13)	0.01 (0-0.02)	-6.74 (-7.22--6.25)
Turkmenistan	0.72 (0.18-1.31)	1.67 (0.39-3.22)	1.32	0.03 (0.01-0.06)	0.03 (0.01-0.07)	-0.95 (-1.75--0.13)
Tuvalu	0.04 (0.01-0.07)	0.02 (0-0.03)	-0.5	0.56 (0.14-1.04)	0.17 (0.04-0.31)	-3.61 (-3.74--3.48)

Uganda	16.27 (3.96-33.97)	23.06 (5.4-46.75)	0.42	0.28 (0.07-0.58)	0.17 (0.04-0.34)	-2.29 (-2.73--1.84)
Ukraine	12.07 (2.79-22.74)	10.67 (2.41-20.54)	-0.12	0.02 (0-0.03)	0.02 (0-0.03)	-0.68 (-1.71-0.36)
United Arab Emirates	0.15 (0.03-0.35)	0.41 (0.08-0.85)	1.73	0.03 (0.01-0.06)	0.02 (0-0.05)	-0.51 (-0.8--0.23)
United Kingdom	2.59 (0.66-4.64)	1.24 (0.31-2.19)	-0.52	0 (0-0)	0 (0-0)	-3.51 (-3.71--3.31)
United Republic of Tanzania	19.14 (4.83-37.58)	30.09 (6.89-61.09)	0.57	0.21 (0.05-0.41)	0.13 (0.03-0.26)	-1.79 (-2.02--1.56)
United States of America	19.08 (4.65-35.02)	7.66 (1.96-13.74)	-0.6	0.01 (0-0.01)	0 (0-0)	-5.39 (-5.95--4.82)
United States Virgin Islands	0.01 (0-0.01)	0.01 (0-0.01)	0	0.01 (0-0.02)	0 (0-0.01)	-2.21 (-2.5--1.92)
Uruguay	0.53 (0.13-0.98)	0.33 (0.08-0.61)	-0.38	0.01 (0-0.03)	0.01 (0-0.01)	-2.41 (-2.78--2.04)
Uzbekistan	3.11 (0.75-5.75)	5.48 (1.32-10.38)	0.76	0.03 (0.01-0.05)	0.02 (0-0.03)	-1.44 (-2.36--0.52)
Vanuatu	0.16 (0.03-0.37)	0.25 (0.06-0.51)	0.56	0.26 (0.06-0.59)	0.14 (0.03-0.29)	-1.98 (-2.06--1.89)
Venezuela (Bolivarian Republic of)	4.02 (1-7.33)	4.18 (0.99-7.66)	0.04	0.04 (0.01-0.08)	0.01 (0-0.03)	-4.23 (-4.77--3.68)
Viet Nam	70.87 (15.82-136.16)	47.75 (11.01-91.89)	-0.33	0.2 (0.04-0.37)	0.05 (0.01-0.1)	-4.13 (-4.19--4.06)
Yemen	3.45 (0.66-7.07)	4.21 (0.89-8.72)	0.22	0.08 (0.02-0.17)	0.03 (0.01-0.07)	-3.16 (-3.41--2.92)
Zambia	12.08 (2.99-22.23)	8.07 (1.71-16.67)	-0.33	0.46 (0.12-0.86)	0.14 (0.03-0.28)	-5.13 (-6.02--4.24)
Zimbabwe	9.43 (2.2-18.37)	24.96 (5.87-49.28	1.65	0.24 (0.06-0.47)	0.36 (0.09-0.69)	2.2 (1.5-2.9

))

Supplementary table 2. The deaths of TB-DLWG cases and rates in 1990 and 2021 across 204 countries, and the trends from 1990 to 2021.
