# Supplementary material for: Global burden of tuberculosis attributable to diet low in whole grains from 1990 to 2021, with projection to 2045
Source: Front Nutr. 2025 Oct 31;12:1679569. doi: 10.3389/fnut.2025.1679569 (PMC12616633; doi:10.3389/fnut.2025.1679569)
Supplement: Supplementary file 2 [file Image_1.pdf]

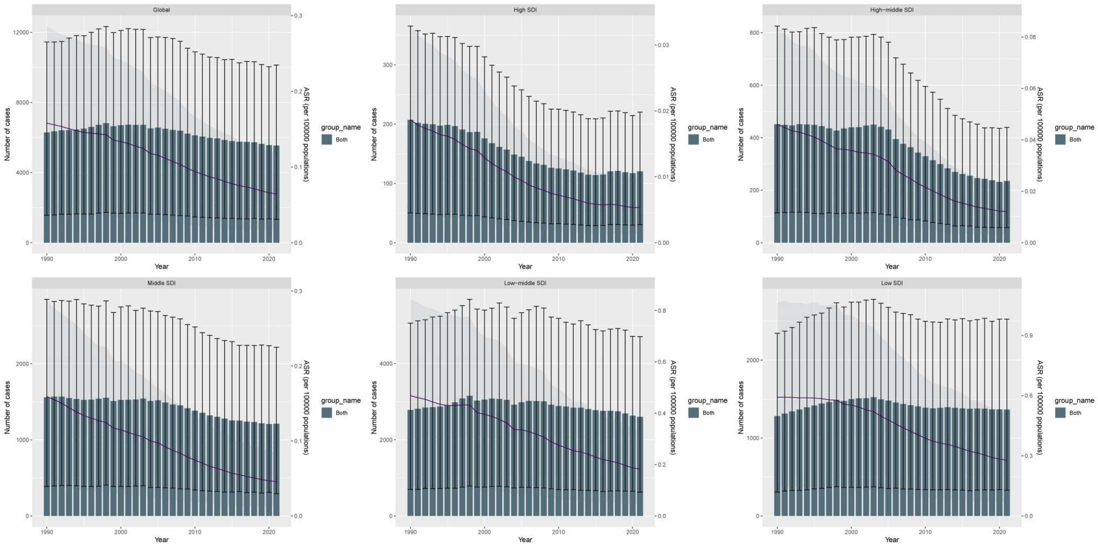

Supplementary figure 1. Death cases and ASMR of TB-DLWG from 1990 to 2021.

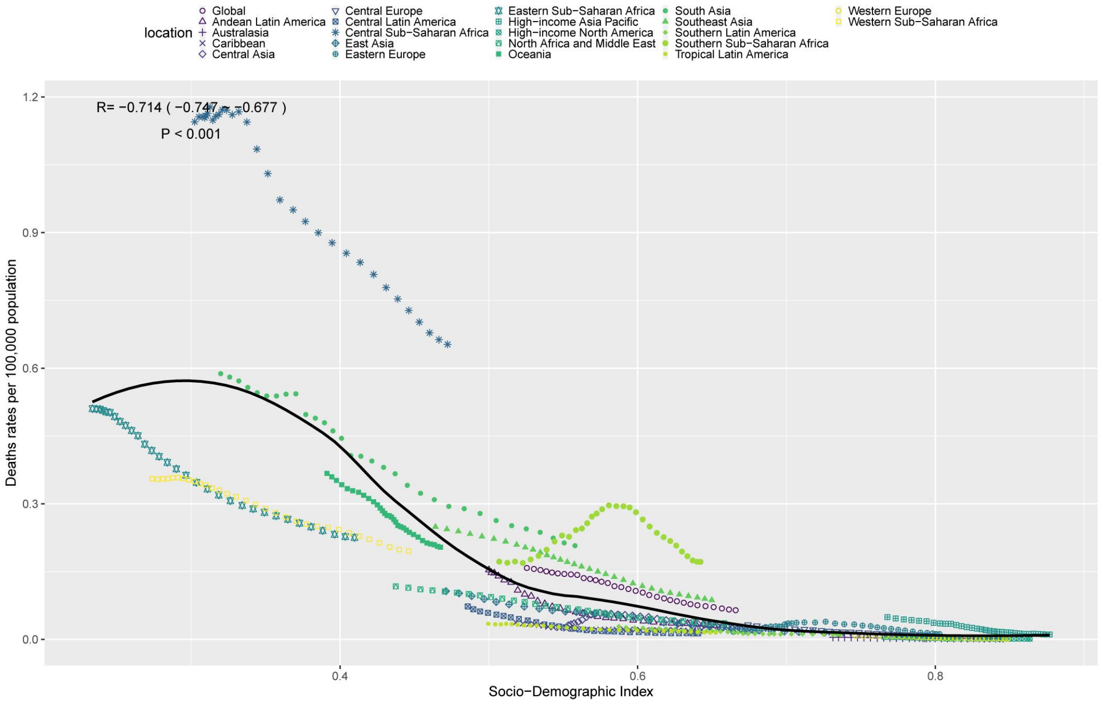

Supplementary figure 2. ASMR of TB-DLWG in 21 GBD regions by SDI, 1990–2021.
